# Supplementary material for: An ultra-conserved poison exon in the Tra2b gene encoding a splicing activator is essential for male fertility and meiotic cell division
Source: EMBO J. 2025 Jan 2;44(3):877–902. doi: 10.1038/s44318-024-00344-6 (PMC11791180; doi:10.1038/s44318-024-00344-6)
Supplement: Supplementary file 1 — Table EV1 [file 44318_2024_344_MOESM1_ESM.docx]

| Protospacer sequence | PAM sequence |
| --- | --- |
| GATCTGTTCAACCCACCCTT | **GGG** |
| GGTGGGTTGAACAGATCTAT | **AGG** |
| GTGGTCTTCTTAATGCCCTT | **TGG** |
| CATGCATTTTGAGAGTCCAA | **AGG** |

**Table EV1.** sgRNAs used to create conditional allele of Tra2b poison exon.
