# Supplementary material for: An ultra-conserved poison exon in the Tra2b gene encoding a splicing activator is essential for male fertility and meiotic cell division
Source: EMBO J. 2025 Jan 2;44(3):877–902. doi: 10.1038/s44318-024-00344-6 (PMC11791180; doi:10.1038/s44318-024-00344-6)
Supplement: Supplementary file 2 — Table EV2 [file 44318_2024_344_MOESM2_ESM.docx]

| Allele | Description |
| --- | --- |
| *Tra2b* | Wild type Tra2b allele |
| *Tra2b^fl^* | Tra2b allele with lox*P*-site-flanked (floxed) exon 4 |
| *Tra2b^-^* | *Tra2b* allele after deletion of exon 4 |
| *Tra2b^fl^*;Vasa-Cre | *Tra2b* allele after deletion of floxed Tra2b exon 4 in germ cells upon Vasa Cre-mediated recombination |
| *Tra2bPE^fl^* | Tra2b allele with lox*P*-site-flanked (floxed) poison exon |
| *Tra2bPE^-^* | *Tra2b* allele after deletion of poison exon |
| *Tra2b*PE*^fl^;Vasa-Cre* | Deletion of floxed Tra2b poison exon in germ cells upon Vasa Cre-mediated recombination |

**Table EV2. Mouse alleles used in this study.**
