# Supplementary material for: An ultra-conserved poison exon in the Tra2b gene encoding a splicing activator is essential for male fertility and meiotic cell division
Source: EMBO J. 2025 Jan 2;44(3):877–902. doi: 10.1038/s44318-024-00344-6 (PMC11791180; doi:10.1038/s44318-024-00344-6)
Supplement: Supplementary file 3 — Table EV3 [file 44318_2024_344_MOESM3_ESM.docx]

| Forward Primer | Reverse Primer | Product | | Product |
| --- | --- | --- | --- | --- |
| Tra2bex4fl F  AGCACCAGCACTCCTCTCTC | **Tra2bex4fl R** ATCGTCTATGCGAGCGAGAT | Floxed allele 489bp | Wild type allele 362bp | |
| Tra2bPEfl F CTAGTCTGTTTATGTGTCCCTGT | **Tra2bPEfl R** ACTAACTGATGGCGAGCTCA | Floxed allele 362bp | | Wild type allele 231bp |
| Tra2bPEwt F AGGGTGGGTTGAACAGATCT | **Tra2bPEwt R** TGTCAAAAGCACATCTCTAGTCA |  |  |  |
| Cre F GAACCTGATGGACATGTTCAGG | **Cre R** AGTGCGTTCGAACGCTAGAGCCTGT | Cre allele 320bp | | Wild type allele 250bp |
| Crecon F TTACGTCCATCGTGGACAGC | **Crecon R**  TGGGCTGGGTGTTAGCCTTA |  |  |  |

**Table EV3**. Primers used for genotyping. Primers Tra2bex4fl F and Tra2bex4fl R were used for genotyping *Tra2b* exon 4 alleles (wild type= 362bp; floxed = 489bp; deleted = 0bp). Primers CreF, CreR, Crecon F and Crecon R were used to detect the presence of Vasa-Cre (320bp detected in mice containing Vasa-Cre transgene). Primers Tra2bPEfl F, Tra2bPEfl R, Tra2bPEwt F, and Tra2bPEwt R were used to genotype *Tra2b PE* alleles (wild type= 231bp; floxed = 362bp; deleted = 0bp).
