# Supplementary material for: An ultra-conserved poison exon in the Tra2b gene encoding a splicing activator is essential for male fertility and meiotic cell division
Source: EMBO J. 2025 Jan 2;44(3):877–902. doi: 10.1038/s44318-024-00344-6 (PMC11791180; doi:10.1038/s44318-024-00344-6)
Supplement: Supplementary file 4 — Table EV4 [file 44318_2024_344_MOESM4_ESM.docx]

| Forward Primer | Reverse Primer | Product | Product |
| --- | --- | --- | --- |
| Ggnbp2F ACACACAAACCTAAACCTCTGG | **Ggnbp2R** CTTCATCCCTGCATTCCTGG | Exon inclusion 202bp | Exon skipped 76bp |
| Slc4a1ap F GATGGAGGTGTCAGGCTTCT | **Slc4a1ap R** TCGGCTCATCTCTGTCTTGT | Exon inclusion 238bp | Exon skipped 151bp |
| Map7d2 F GGAGAGGCTGCAAAGATCCT | **Map7d2 R1** CTTTCCTTTCTCACCTTGCG  **Map7d2 R2** TTCTCTTCTTCCTCCTGCCG | Exon inclusion 145bp | Exon skipped 203bp |
| Rpgr F TGATGACTACGAGTTCCAATGAG | **Rpgr R1** ATGCTTCCAGAATCTCGGCT  **Rpgr R2** TCTCCTTCTCTCCTTCCTTCC | Exon inclusion 222bp | Exon skipped 174bp |
| Ptbp2 F TGATCTGCCATCTGGAGACG | **Ptbp2 R1** GATTACAGACTGGTGTCATG  **Ptbp2 R2** ATTGCTAACCAACAGGAC | Exon inclusion 118bp | Exon skipped 208bp |
| Phf7 F ACCGAGAAGAGTTCCCACAG | **Phf7 R** GATCCACACGTAGCACACAA | Exon inclusion 208bp | Exon skipped 91bp |
| Tra2a F  GTTGTAGCCGTCGCCTTCT | **Tra2a poison R1**  TTCAAGTGCTTCTATCTGACCAA  **Tra2a R2**  TGGGATTCAGAATGTTTGGA | Exon inclusion 303bp | Exon skipped 258bp |
| Tra2b F  GAGCTCCTCGCAAAAGTGTG | **Tra2b poison R1**  CATCTTCCCCACTTCACACA  **Tra2b R2**  CAACATGACGCCTTCGAGTA | Exon inclusion 331bp | Exon skipped 438bp |

**Table EV4.** Primers used to detect alternative mRNA isoforms in P12 mouse testis.
