# Supplementary material for: An ultra-conserved poison exon in the Tra2b gene encoding a splicing activator is essential for male fertility and meiotic cell division
Source: EMBO J. 2025 Jan 2;44(3):877–902. doi: 10.1038/s44318-024-00344-6 (PMC11791180; doi:10.1038/s44318-024-00344-6)
Supplement: Supplementary file 5 — Table EV5 [file 44318_2024_344_MOESM5_ESM.docx]

| Forward primer | Reverse primer | Exon inclusion amplicon size (bp) | Exon exclusion amplicon size (bp) |
| --- | --- | --- | --- |
| mSrsf3PE F CTGCCGTGTAAGAGTGGAAC | **mSrsf3PE R1** GAGAAGGATCGAGACGGCTT  **mSrsf3PE scrR2** ATGCAGATTCAGAGGGTGGT | 165 | 237 |
| mSrsf4PE234 F CCTGGAGGTGGATCTGAAGA | **mSrsf4PE234 R** GGTCTTTGCCGTTCAGTTCA  **mSrsf4PE scr2R**  GGTCTCAGGGTAGGGTGAAG | 163 | 95 |
| mSrsf5PE F ATCCTCAAGAGTCAGCTGGC | **mSrsf5PE R** TCCCCAGCTTGTCTCATGAA | 189 | 54 |
| mSrsf6PE F CCGCGCGTCTACATAGGA | **mSrsf6PE R** ACTGCTGTATCCACCTCCAC | 441 | 276 |
| mSrsf7PE F TAGCCGACGAAGAAGAAGCA | **mSrsf7PE R** GTATCGCCTTCCCCTGGATC | 524 | 64 |
| mSrsf9PE F GTTCCCCAGGACTTACGGAG | **mSrsf9PE R** ACAGACATCCCCAGCTTCTC | 250 | 160 |
| mSrsf10PE F ATTTCTACACTCGGCGTCCA  mSrsf10PE scrF TGGGAGACAAAGCCTCGATT | **mSrsf10PE R** CTGACGCCCACAAATCCATT | 225 | 110 |
| mSrsf11PE F TGTGTTCGTTGACAGAGCTT | **mSrsf11PE R** GCTGGTGCCAATAGAGACAA | 199 | 90 |
| Tra2b PE F  GAGCTCCTCGCAAAAGTGTG | **Tra2b PE R**  CAACATGACGCCTTCGAGTA | 714 | 438 |
| Tra2a PE F  GTTGTAGCCGTCGCCTTCT | **Tra2a PE R**  TTCAAGTGCTTCTATCTGACCAA  **Tra2a PE R1**  TGGGATTCAGAATGTTTGGA | 303 | 258 |

**Table EV5.** Primers used to screen poison exons of SR protein family genes.
