# Supplementary material for: An ultra-conserved poison exon in the Tra2b gene encoding a splicing activator is essential for male fertility and meiotic cell division
Source: EMBO J. 2025 Jan 2;44(3):877–902. doi: 10.1038/s44318-024-00344-6 (PMC11791180; doi:10.1038/s44318-024-00344-6)
Supplement: Supplementary file 6 — Table EV6 [file 44318_2024_344_MOESM6_ESM.docx]

| Target | Forward primer | Reverse primer | Amplicon size (bp) |
| --- | --- | --- | --- |
| *Tra2a* | CCATCACGGGTGTCCAAAC | TGAGAGTGTGACCTGGATCG | 113 |
| *Malat1* | GTACTGTATAATGGAGGCTG | GCCGACCTTCAAACTAGAACC | 91 |
| *Ptbp2* | TCTCAGGCAGTGTTCTCAGC | GCCCCATCCATTTTATCTTCTCC | 109 |
| *Rbpms2* | GTTTGTCAGTGGCCTCCCT | AACAGGCTGTCTTGAGGTGA | 112 |
| *Mael1* | GCATGACCAAGCAACTGTGT | CGCTCCATACGCTTCAAACA | 129 |
| *Rad51* | TACATTGACACCGAGGGCAC | GTTGAACCCTCGCGCATATG | 114 |
| *Mei1* | GTACAGTCTTTCCCGCCAAC | CTTTCGGGCTGTATCCACG | 116 |
| *Tra2B* | AGACGTTCAAGAGGATTTGCC | TAATTCGACGCCCATCAAGC | 103 |
| *Slc25a31* | AGATTTTGCCAGAACCCGAT | TAATGCAGTCACCCAAACCC | 83 |
| *Majin* | TTCATGCAGGACCCAATGTG | AGCTCCTGGACGATGACTTC | 97 |
| *ActB* | TGACGTTGACATCCGTAAAG | GAGGAGCAATGATCTTGATCT | 143 |
| *Gapdh* | GGCTGCCCAGAACATCATCC | GTCATCATACTTGGCAGGTTTCTC | 169 |
| *Hprt* | CAAACTTTGCTTTCCCTGGT | TCTGGCCTGTATCCAACACTTC | 101 |

**Table EV6.** Primers used in RT-qPCR
