# Supplementary material for: An ultra-conserved poison exon in the Tra2b gene encoding a splicing activator is essential for male fertility and meiotic cell division
Source: EMBO J. 2025 Jan 2;44(3):877–902. doi: 10.1038/s44318-024-00344-6 (PMC11791180; doi:10.1038/s44318-024-00344-6)
Supplement: Supplementary file 7 — Appendix [file 44318_2024_344_MOESM7_ESM.pdf]

Appendix Figures

# An ultra-conserved poison exon in Tra2b is essential for male fertility and meiotic cell division

Contents

Page 1 Contents
Page 2 Appendix Figure S1 The *Tra2b* poison exon is highly expressed in adult testis, and upregulated within spermatocytes
Page 3. Appendix Figure S2. Downregulation of genes within Tra2b cPEko germ cells known to be important in meiosis.
Page 4 Appendix Figure S3. Global analysis of gene expression and splice differences.
Page 5 Appendix Figure S4. Confirmation of splicing changes within independently collected P14 testis RNA samples and with –RT control.
Page 6 Appendix Figure S5. Inclusion of the Tra2b PE is reduced in Tra2b-cPEko P12 and P14 mouse testes.
Page 7 Appendix Figure S6. *Malat1* is strongly bound by Tra2β.

**Appendix Figure 1**

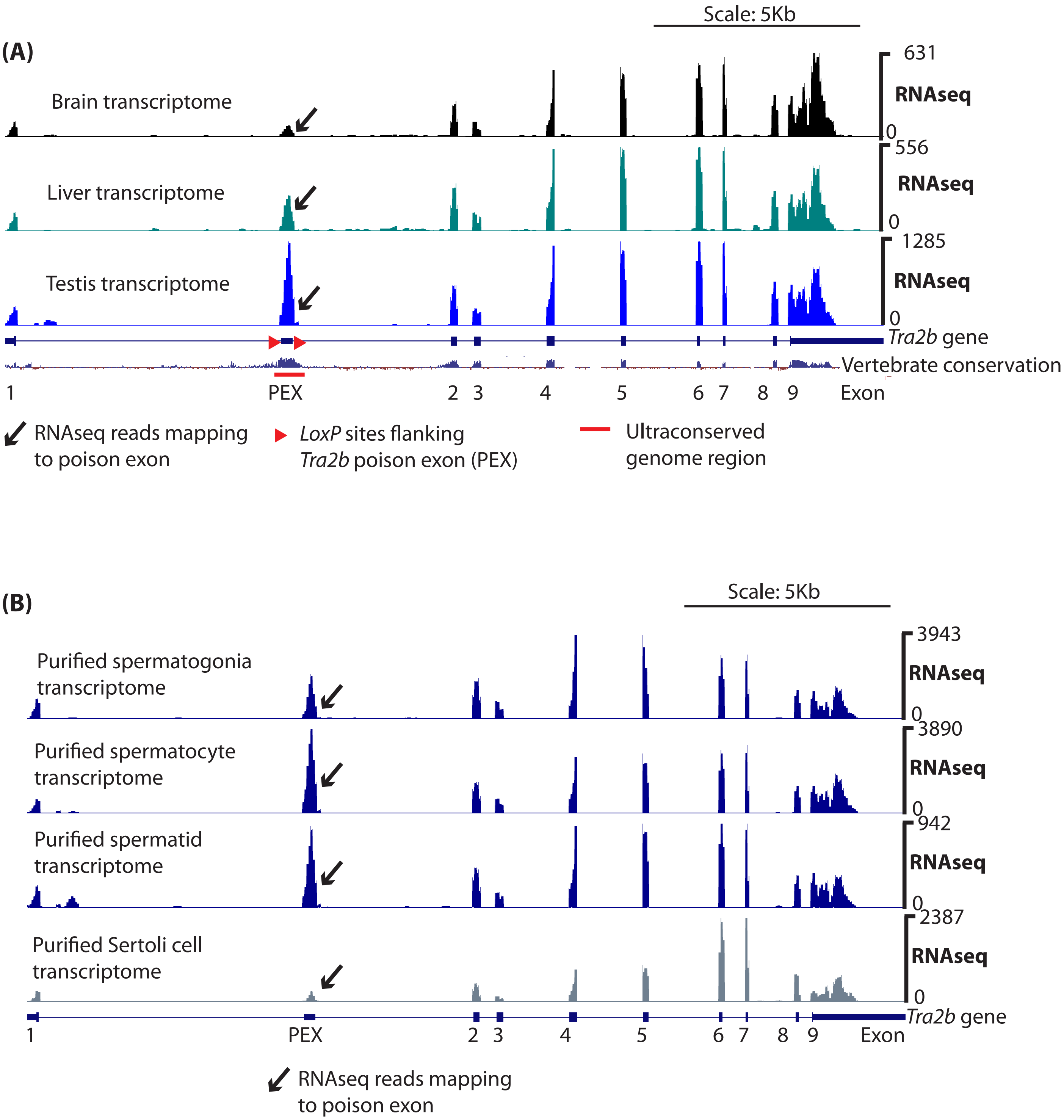


Appendix Figure S1. The *Tra2b* poison exon is highly expressed in adult testis, and upregulated within spermatocytes. Publicly available mRNAseq data from (A) adult mouse tissues (GEO accession GSE43721)and (B) purified germ cell types (GEO accession GSE43717) was aligned with the mouse genome mm39, and visualised on the UCSC genome browser.

(A)

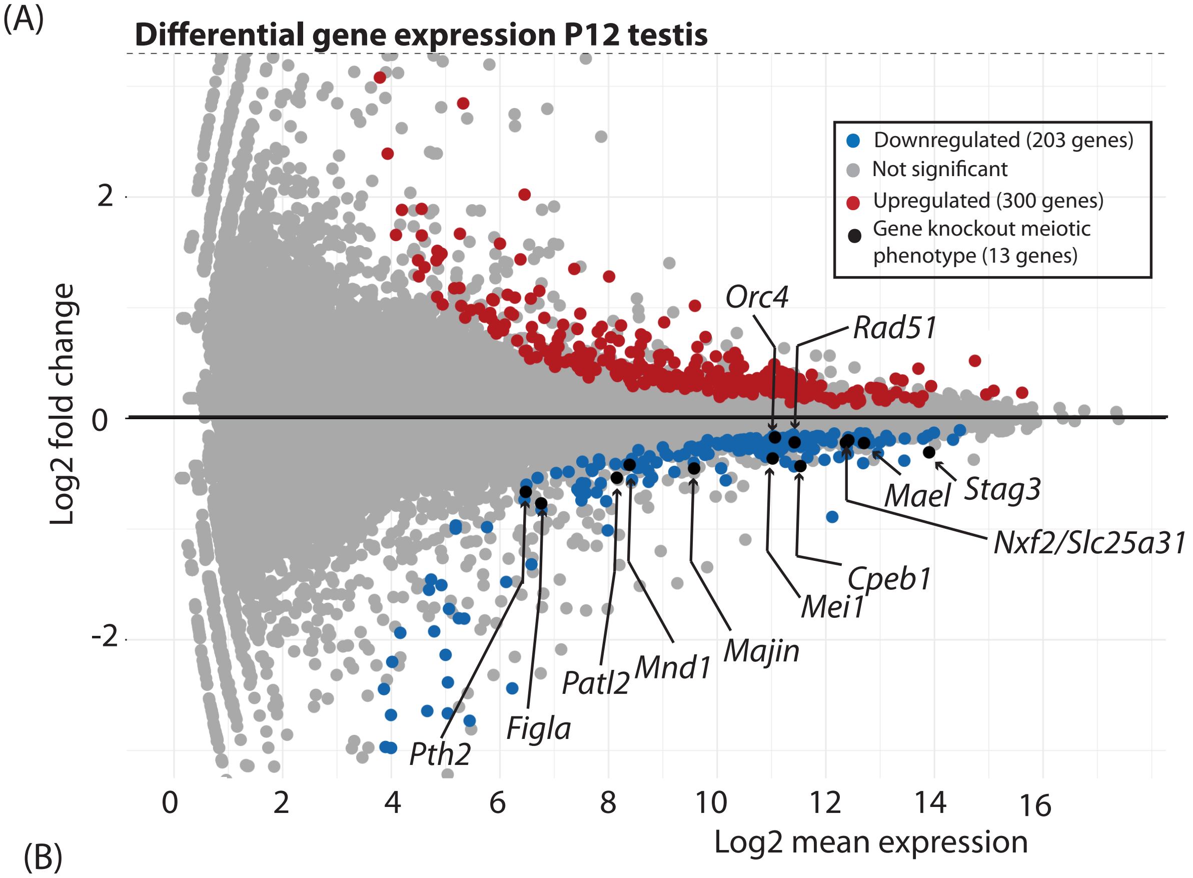


(B)

| Gene name | Log2 fold change | Padjust | Function |
|---|---|---|---|
| *Stag3* | -0.30662 | 0.025286 | Subunit of cohesin complex |
| *Mael* | -0.2226 | 0.03862 | Transposon silencer required for chromosome synapsis |
| *Nxf2* | -0.19696 | 0.036064 | Nuclear RNA export factor |
| *Slc25a31* | -0.21909 | 0.038749 | mitochondrial ADP/ATP carrier protein |
| *Cpeb1* | -0.43096 | 0.005598 | Post-transcriptional gene expression regulator |
| *Rad51* | -0.21635 | 0.034748 | Recombinase involved in homologous recombination |
| *Orc4* | -0.17167 | 0.044508 | Required for initiation of DNA replication |
| *Mei1* | -0.36205 | 0.005915 | Meiotic double strand break formation protein |
| *Majin* | -0.45232 | 0.0453 | Involved in homologous chromosome pairing |
| *Mnd1* | -0.4197 | 0.020604 | Binds DNA and stimulates activity of RAD51 |
| *Fig1a* | -0.76763 | 0.009311 | Transcription factor |
| *Pth2* | -0.66368 | 0.045405 | Neuropeptide |

Appendix Figure S2. Downregulation of genes within Tra2b cPEko germ cells known to be important in meiosis. Panel (A) shows the same MA plot as in Fig.2, but with genes labelled where an individual knockout is reported to cause a male meiotic defect on the MGI browser (https://www.informatics.jax.org/batch). Panel (B) shows a table of these genes with functions briefly annotated from GeneCards (https://www.genecards.org/).

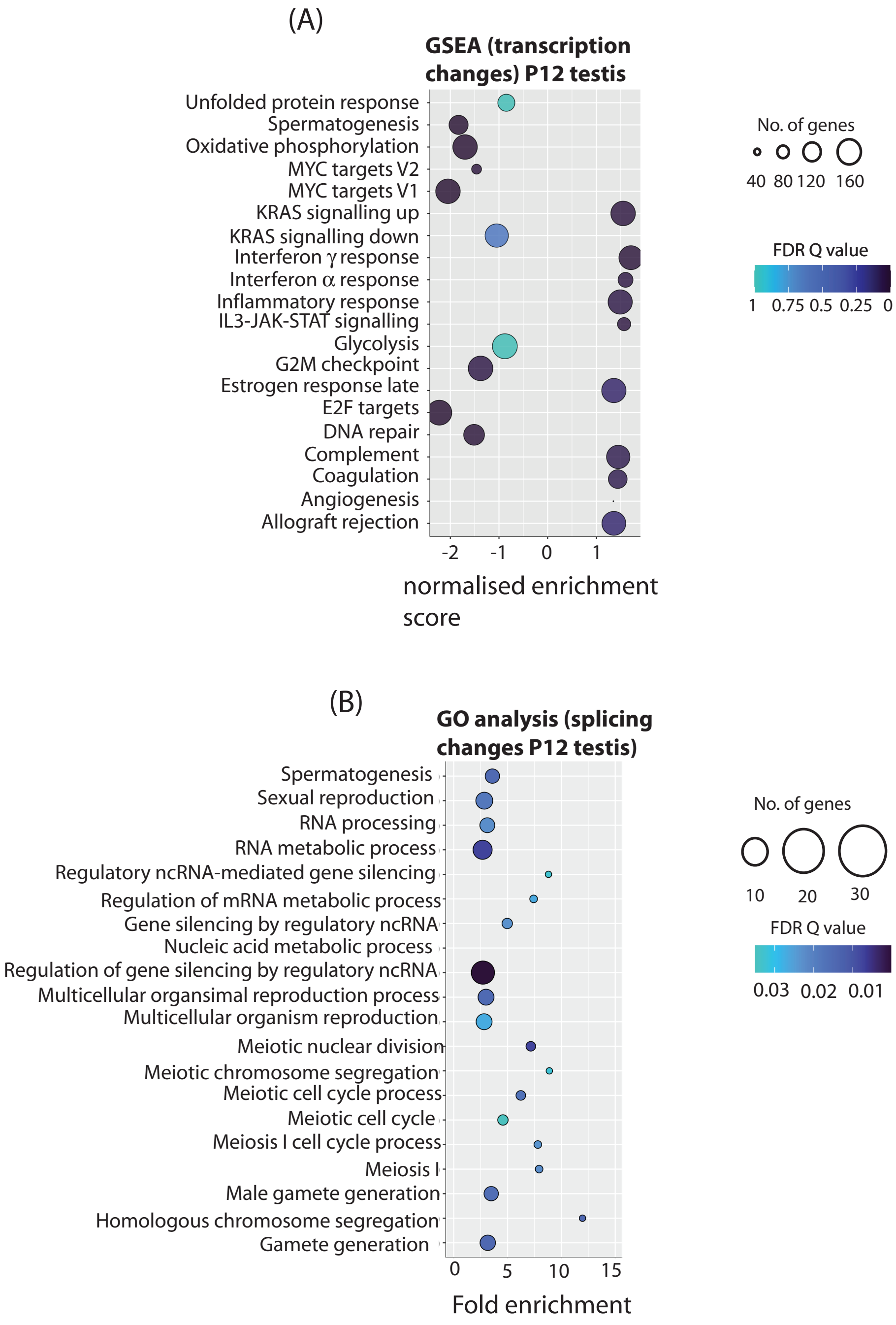


Appendix Figure 3. Global analysis of gene expression and splice differences.
(A) Gene Set Enrichment Analysis (GSEA) of the 504 genes that are differentially expressed between P12 testes from wild type and Tra2b-cPEko mice.
(B) Gen Ontology (GO) analysis of the 157 genes that with predicted splice changes between wild type and Tra2b-cPEko mice.

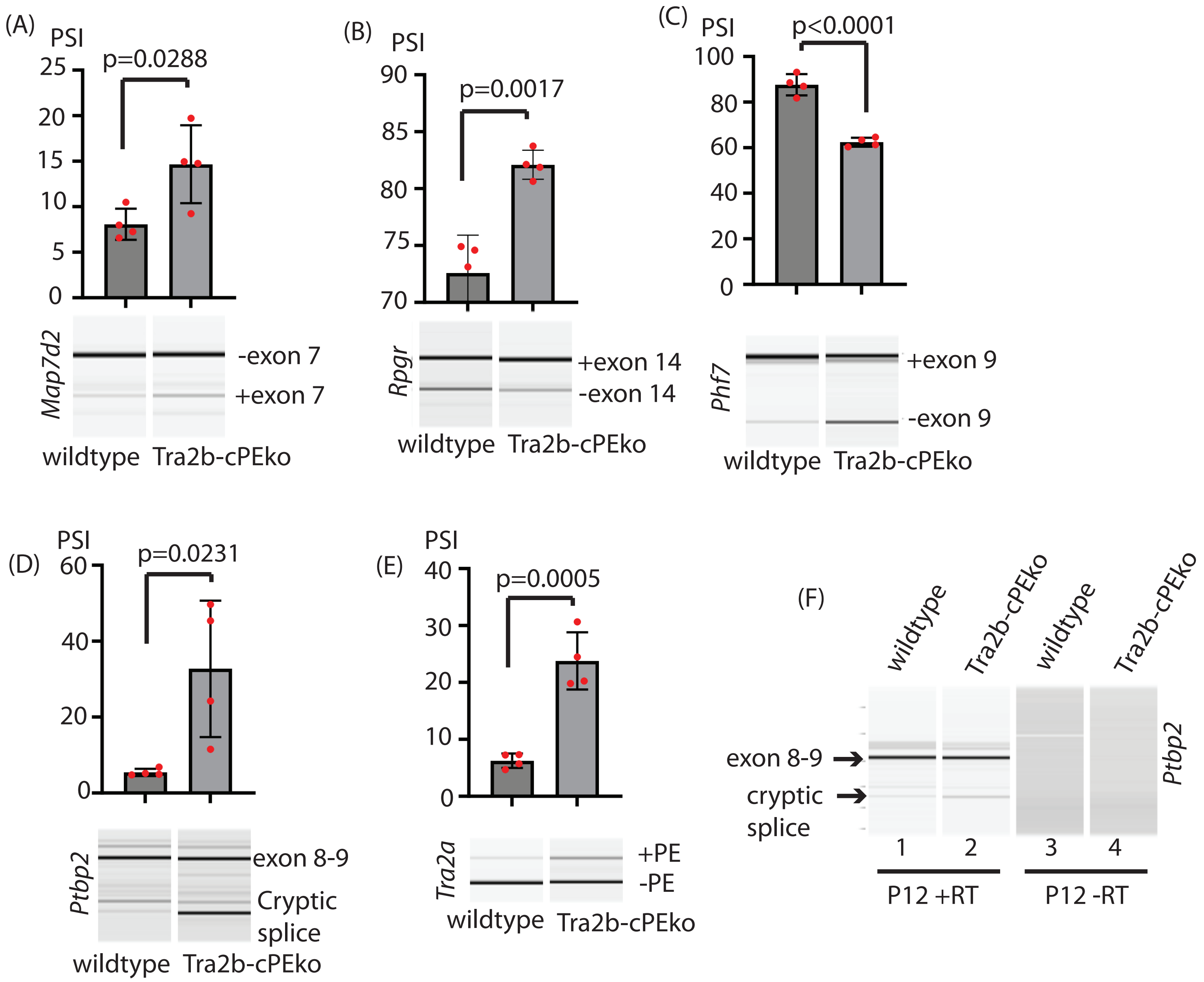


Appendix Figure 4. Confirmation of splicing changes within independently collected P14 testis RNA samples and with –RT control. (A-E) Splicing patterns in whole P14 testis were monitored by RT-PCR as in Figures 3 and 4, and analysed by capillary gel electrophoresis. Example lanes for each genotype are shown in the lower panels, and quantifications in the upper panels. The mean PSI values and standard deviations are shown for n=4 biologically individual wild type testes and n=4 individual Tra2b-cPEko testes (values for individual samples are shown as red dots). P values were calculated by T tests. (F) Analysis of P12 whole testis RNA by RT-PCR to detect use of the Ptbp2 cryptic splice site. The experiment was run in parallel with reverse transcriptase (lanes 1 and 2) and minus reverse transcriptase (lanes 3 and 4).

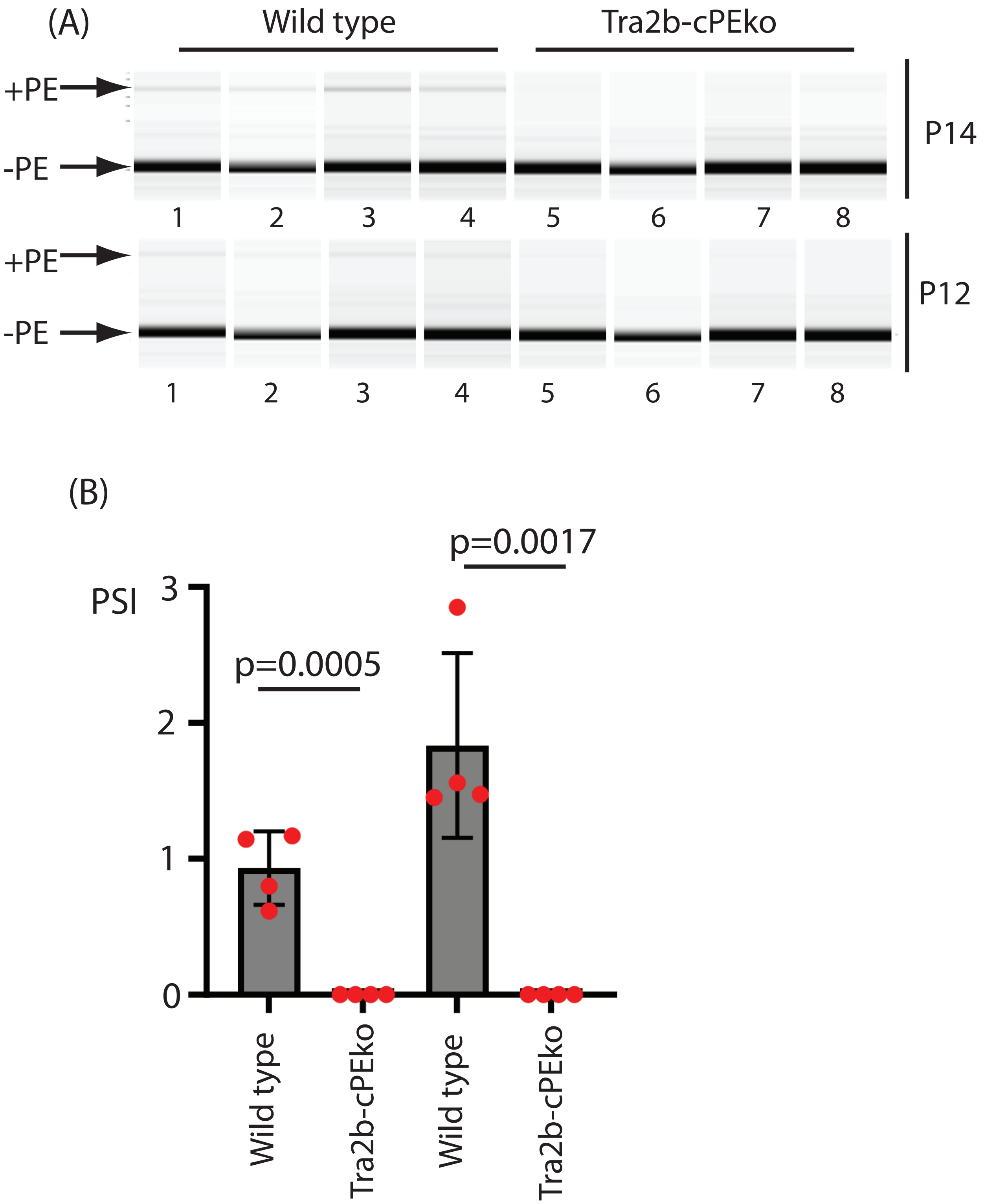


Appendix Figure S5. Inclusion of the Tra2b PE is reduced in Tra2b-cPEko P12 and P14 mouse testes. (A) Capillary gel electrophoresis of RT-PCR reaction products using primers within Tra2b exons 1 and 2 (so detecting Tra2b poison exon inclusion as an additional larger band). Analyses were carried out on n=4 P12 wild type and n=4 Tra2b-cPEko whole testes (using samples used for RNAseq), and n=4 of each genotype from P14 whole testes. (B) Plot showing calculated PSI values from these electrophoretograms analysing RNA from P12 and P14 whole testes (individual samples shown as red dots). Statistical significance between genotypes at P12 and P14 were calculated using T tests.

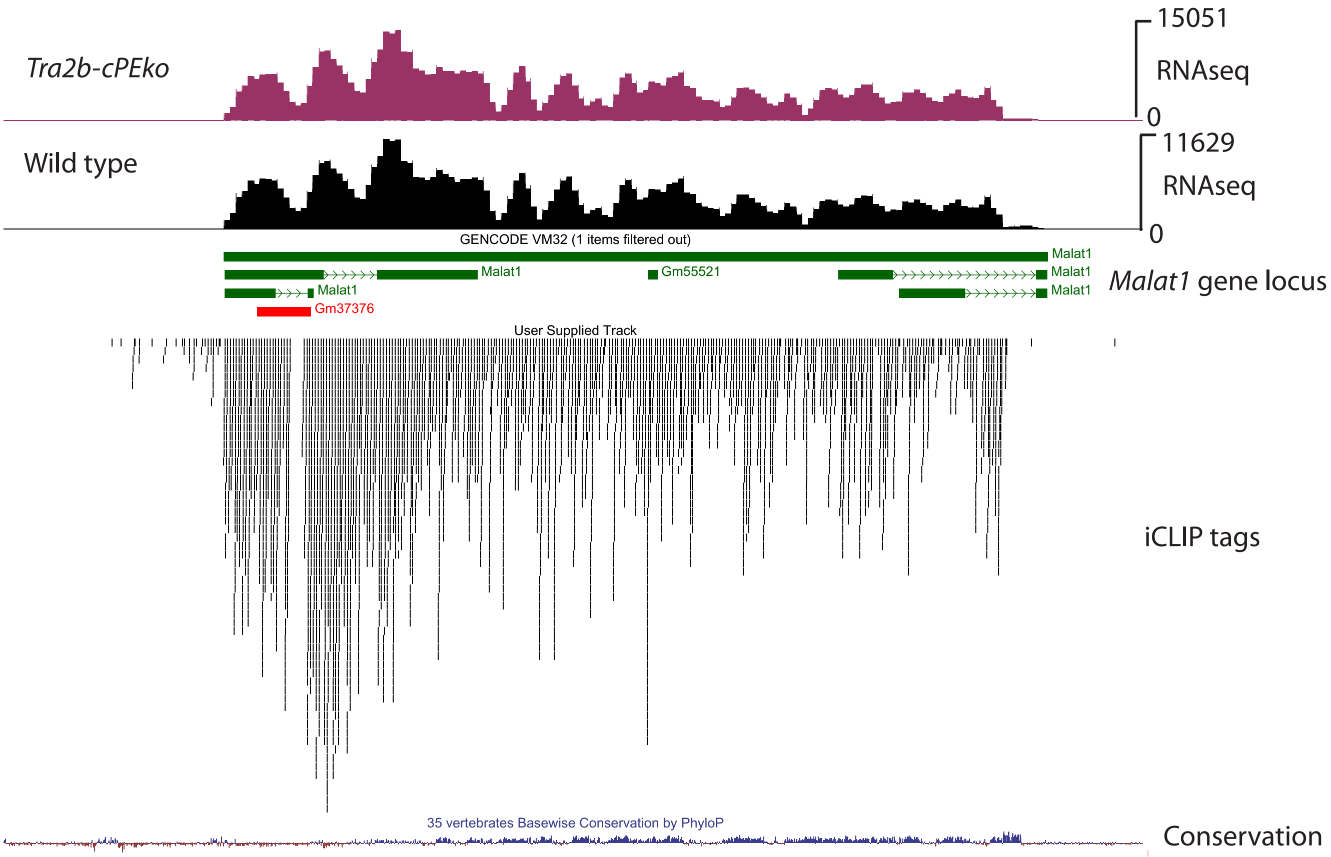


Appendix Figure S6 *Malat1* is strongly bound by Tra2β. UCSC genome browser screenshot showing iCLIP reads aligned to the mouse genome (mm39) at the Malat1 locus.
