# Supplementary figures and images for: An ultra-conserved poison exon in the Tra2b gene encoding a splicing activator is essential for male fertility and meiotic cell division
Source: EMBO J. 2025 Jan 2;44(3):877–902. doi: 10.1038/s44318-024-00344-6 (PMC11791180; doi:10.1038/s44318-024-00344-6)

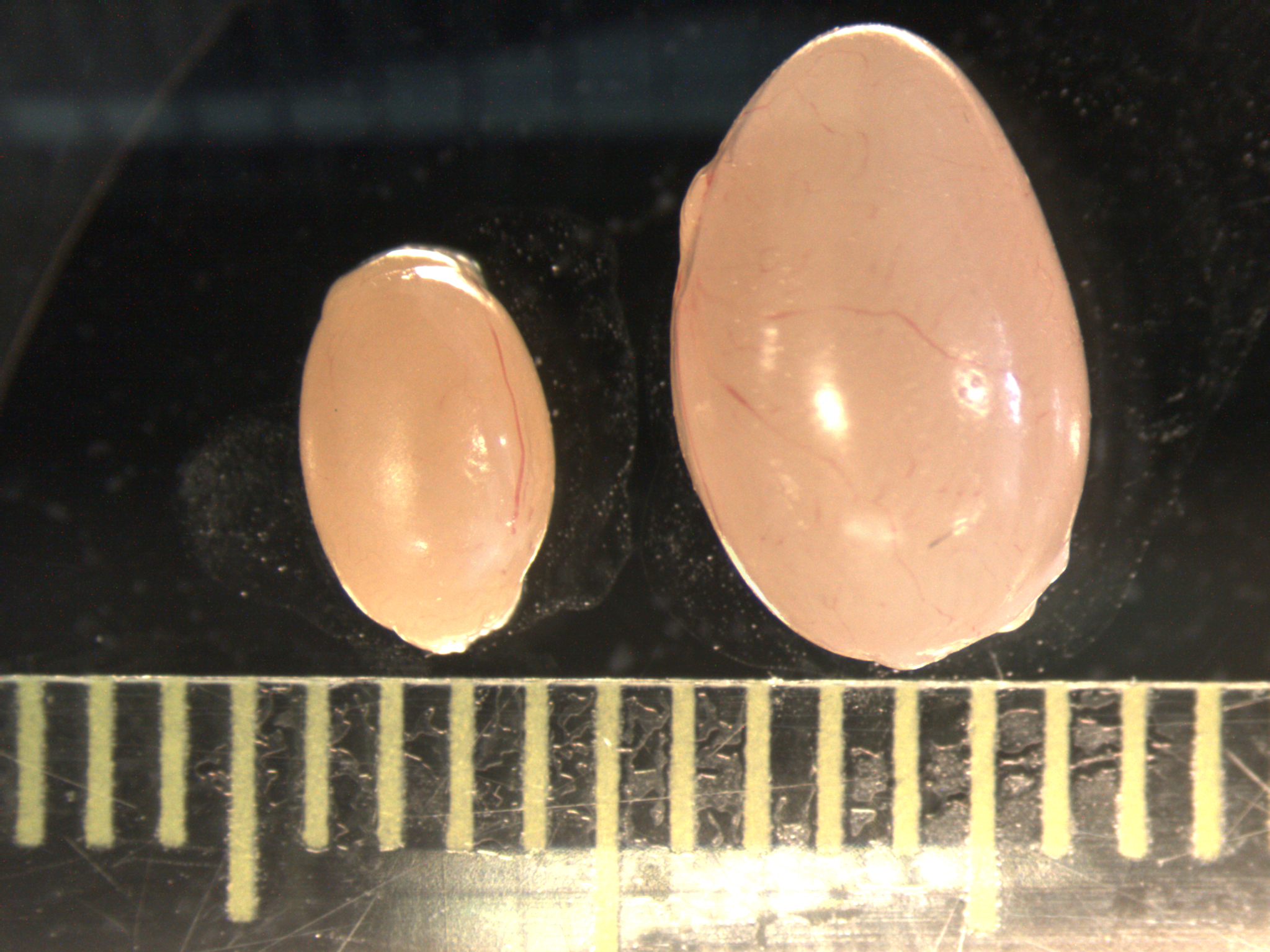

Supplement: Supplementary file 9 — Source data Fig. 1 [file 44318_2024_344_MOESM9_ESM.zip › Figure 1/Fig 1D original image.tif]

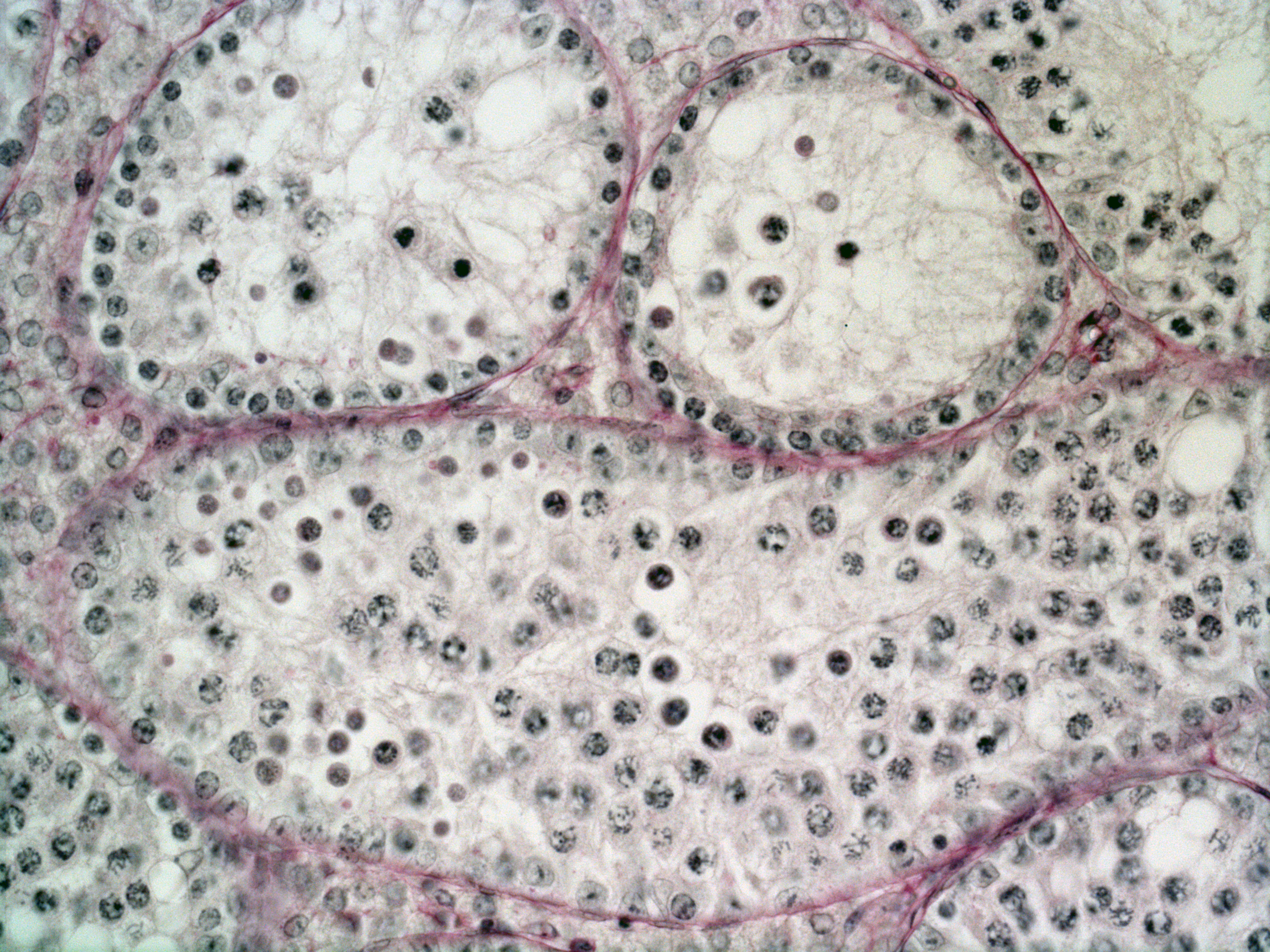

Supplement: Supplementary file 9 — Source data Fig. 1 [file 44318_2024_344_MOESM9_ESM.zip › Figure 1/Figure 1H original image.tif]

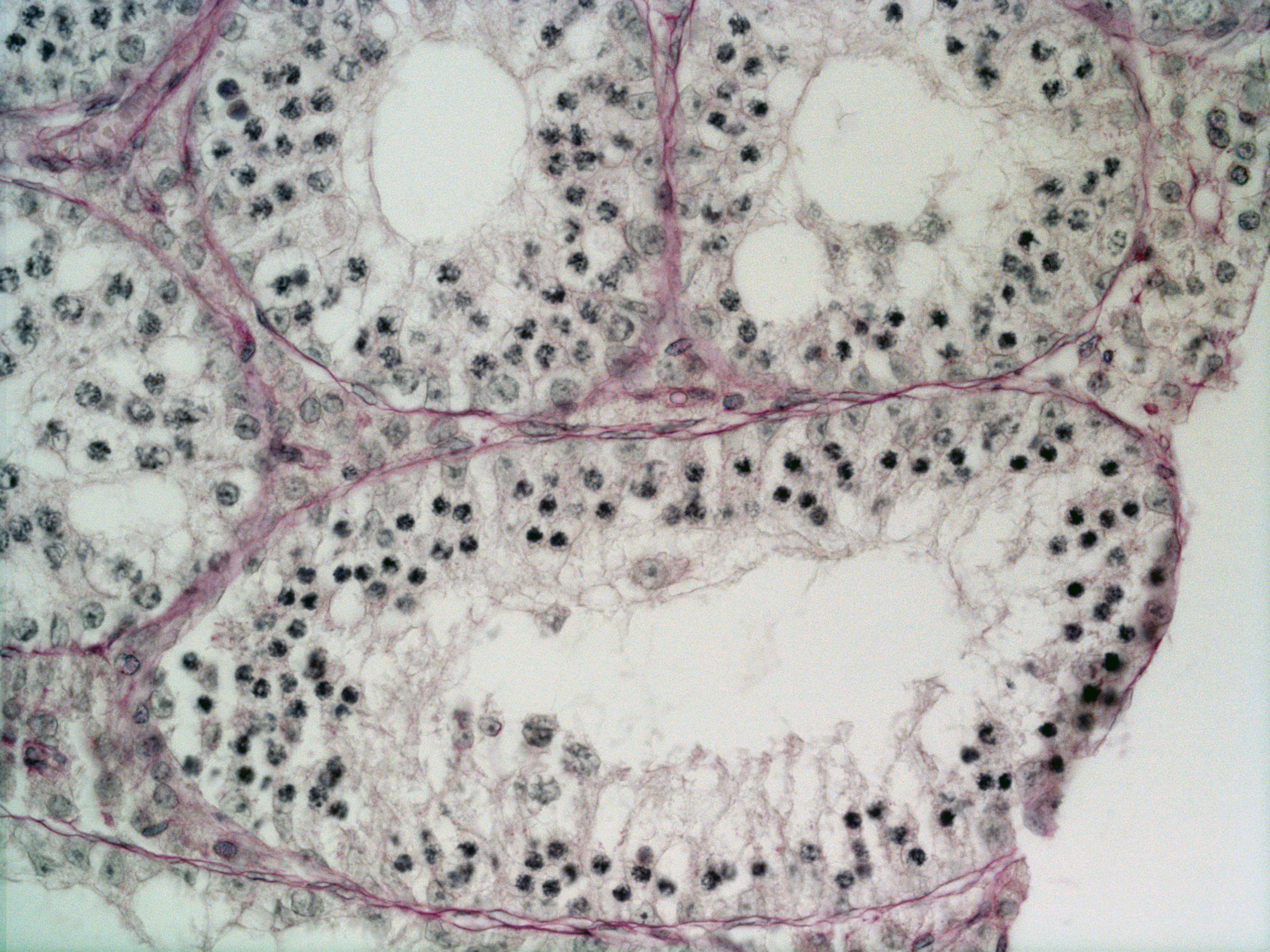

Supplement: Supplementary file 9 — Source data Fig. 1 [file 44318_2024_344_MOESM9_ESM.zip › Figure 1/Figure 1I original image.tif]

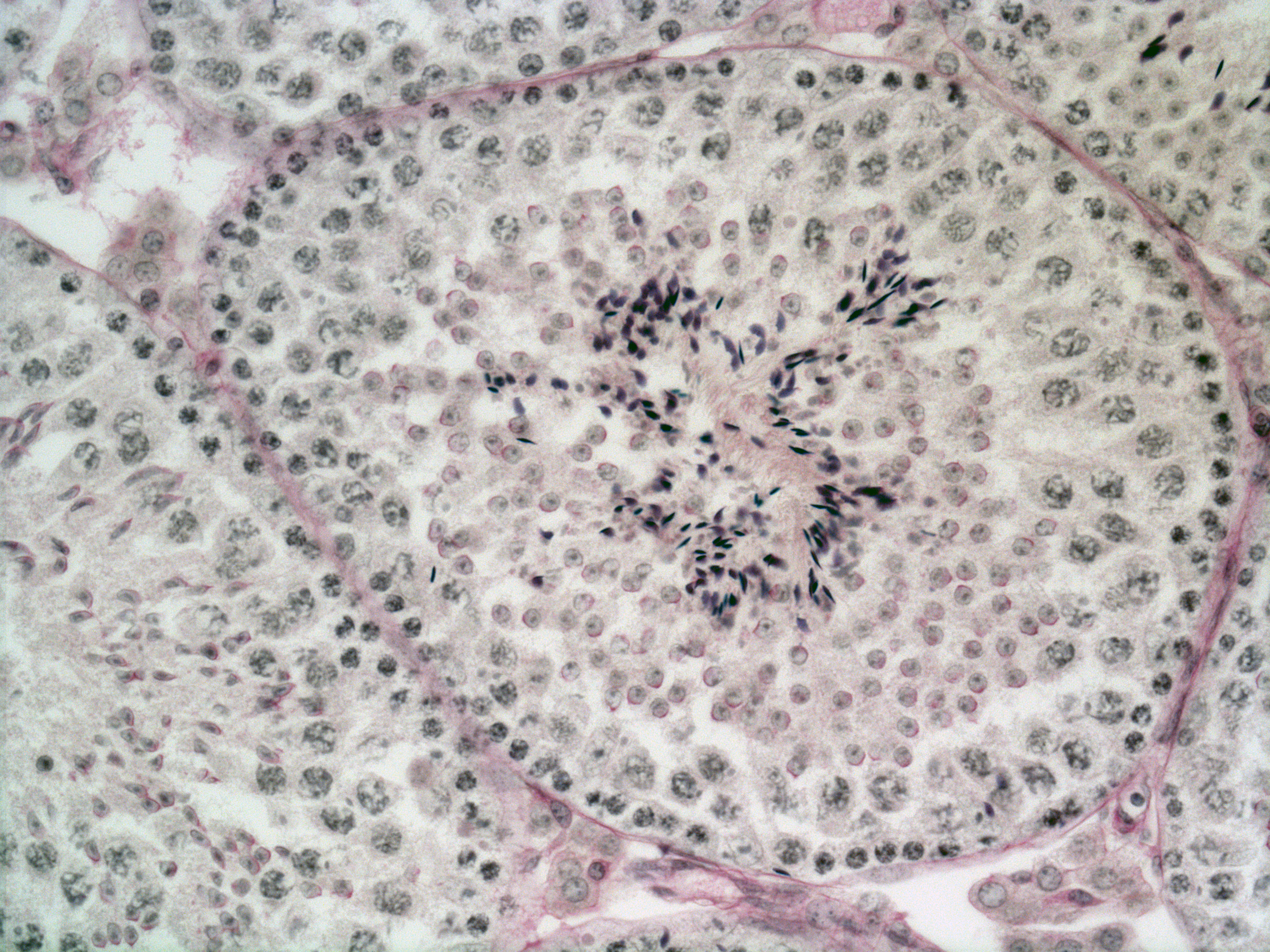

Supplement: Supplementary file 9 — Source data Fig. 1 [file 44318_2024_344_MOESM9_ESM.zip › Figure 1/Figure 1G original image.tif]

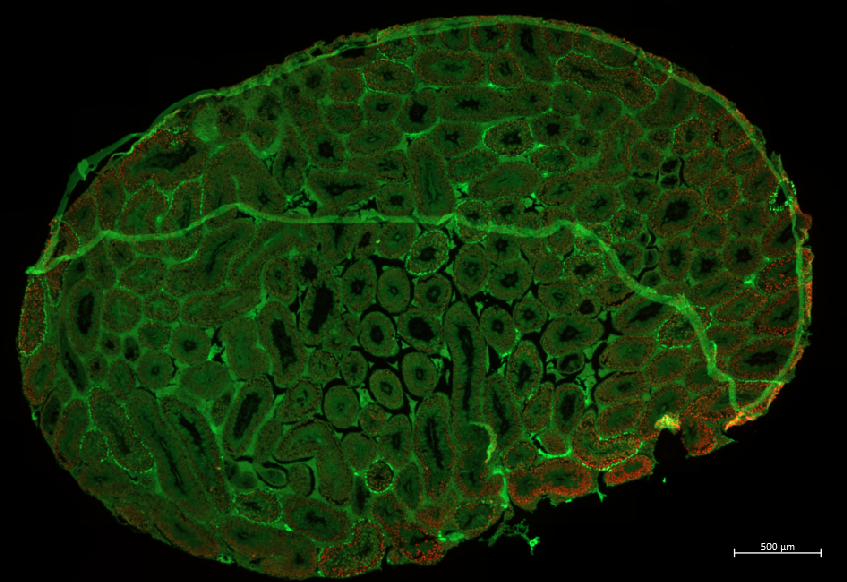

Supplement: Supplementary file 10 — Source data Fig. 2 [file 44318_2024_344_MOESM10_ESM.zip › Figure 2/Fig 2A/adultWT_tiles_20x.tif]

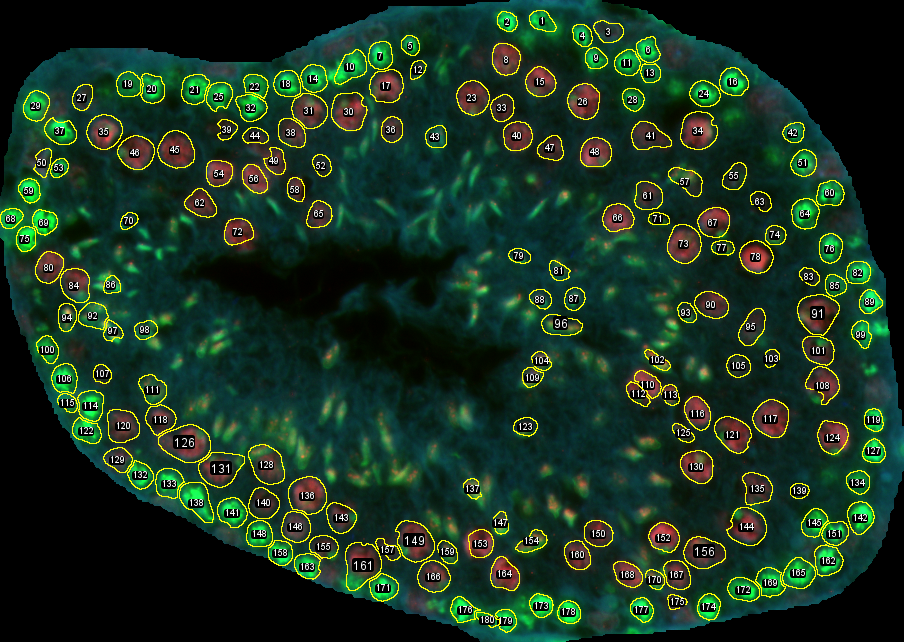

Supplement: Supplementary file 10 — Source data Fig. 2 [file 44318_2024_344_MOESM10_ESM.zip › Figure 2/Fig 2B and 2C/RGB_Image_Plus_Cell_Overlay_With_Numbers.png]

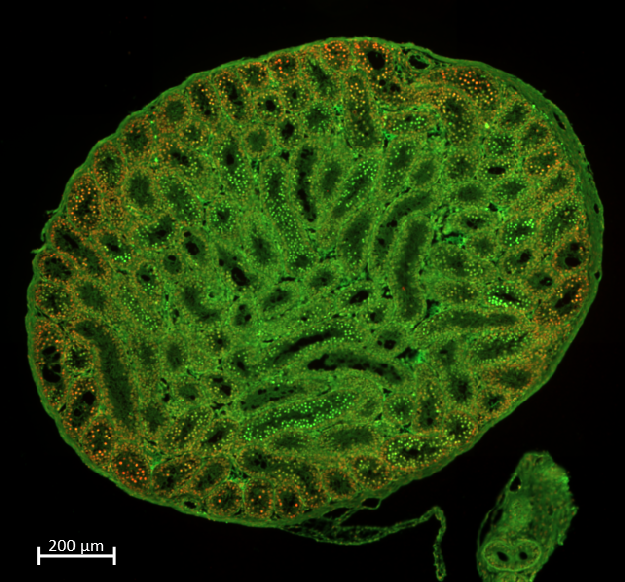

Supplement: Supplementary file 11 — Source data Fig. 3 [file 44318_2024_344_MOESM11_ESM.zip › Figure 3/Fig 3C/p12_PE1_1_KO_tiles_20x.tif]

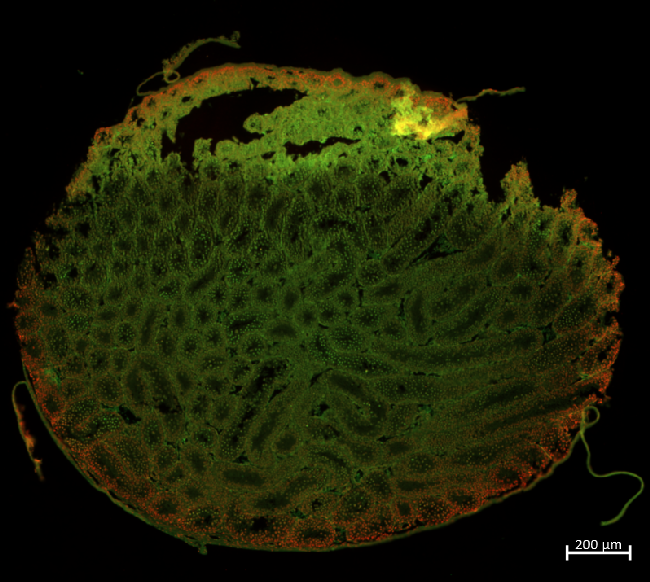

Supplement: Supplementary file 11 — Source data Fig. 3 [file 44318_2024_344_MOESM11_ESM.zip › Figure 3/Fig 3C/p12_WT_tiles_20x.tif]

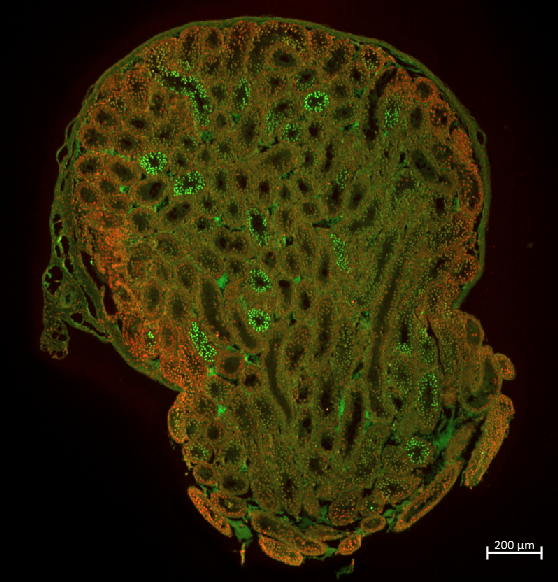

Supplement: Supplementary file 11 — Source data Fig. 3 [file 44318_2024_344_MOESM11_ESM.zip › Figure 3/Fig 3C/p12_TF18_het_tiles_20x.tif]

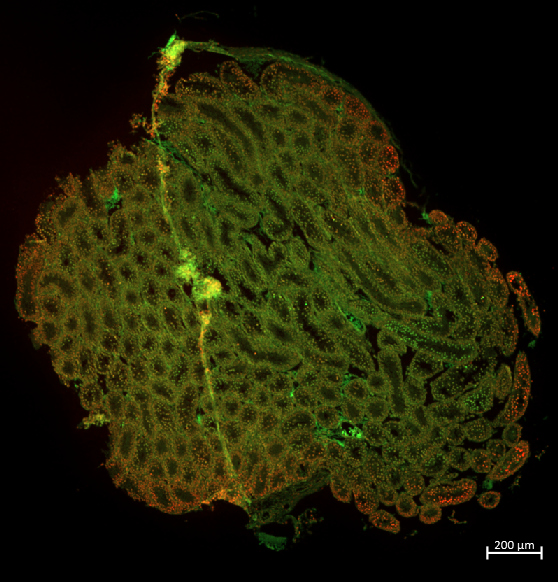

Supplement: Supplementary file 11 — Source data Fig. 3 [file 44318_2024_344_MOESM11_ESM.zip › Figure 3/Fig 3C/p12_KO_tiles_20x.tif]

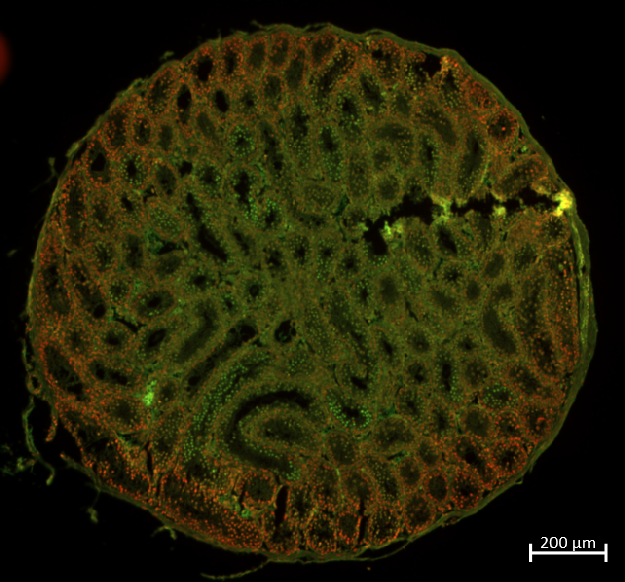

Supplement: Supplementary file 11 — Source data Fig. 3 [file 44318_2024_344_MOESM11_ESM.zip › Figure 3/Fig 3C/p12_PE1_3_het_tiles_20x.tif]

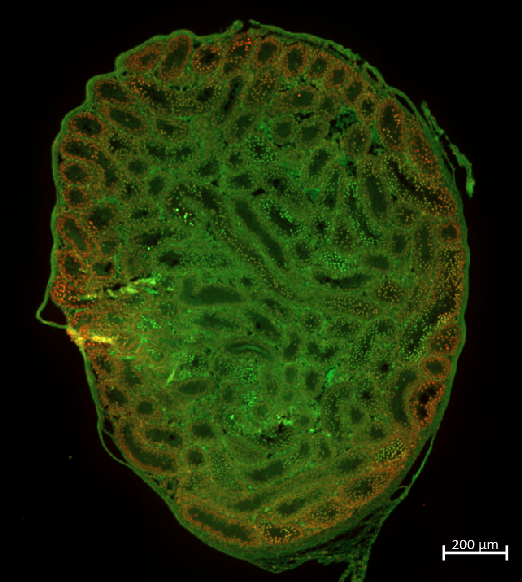

Supplement: Supplementary file 11 — Source data Fig. 3 [file 44318_2024_344_MOESM11_ESM.zip › Figure 3/Fig 3C/p12_PE1_2_KO_tiles_20x.tif]

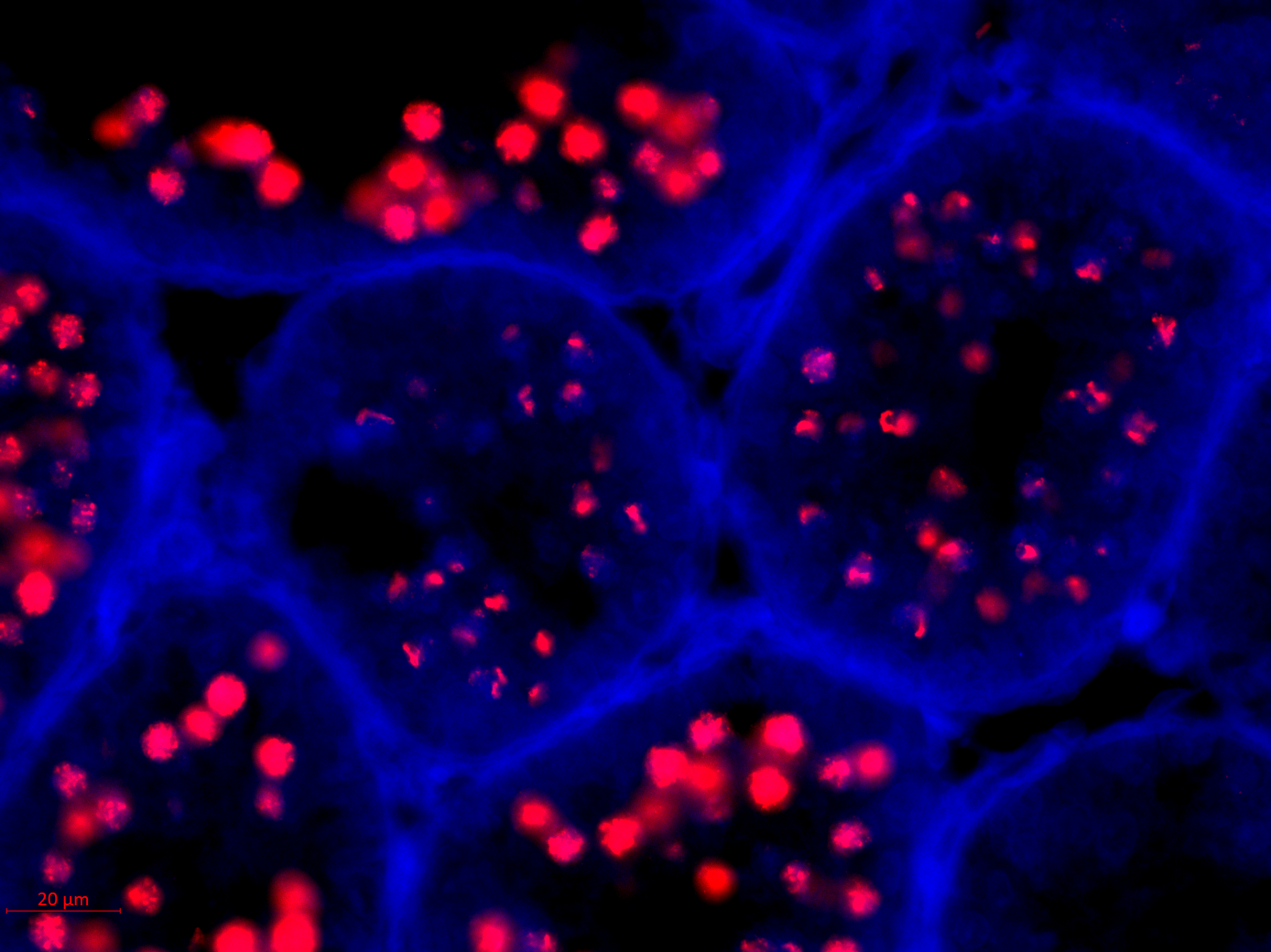

Supplement: Supplementary file 11 — Source data Fig. 3 [file 44318_2024_344_MOESM11_ESM.zip › Figure 3/Fig 3A/Fig 3A Tra2b-cPEKO.tif]

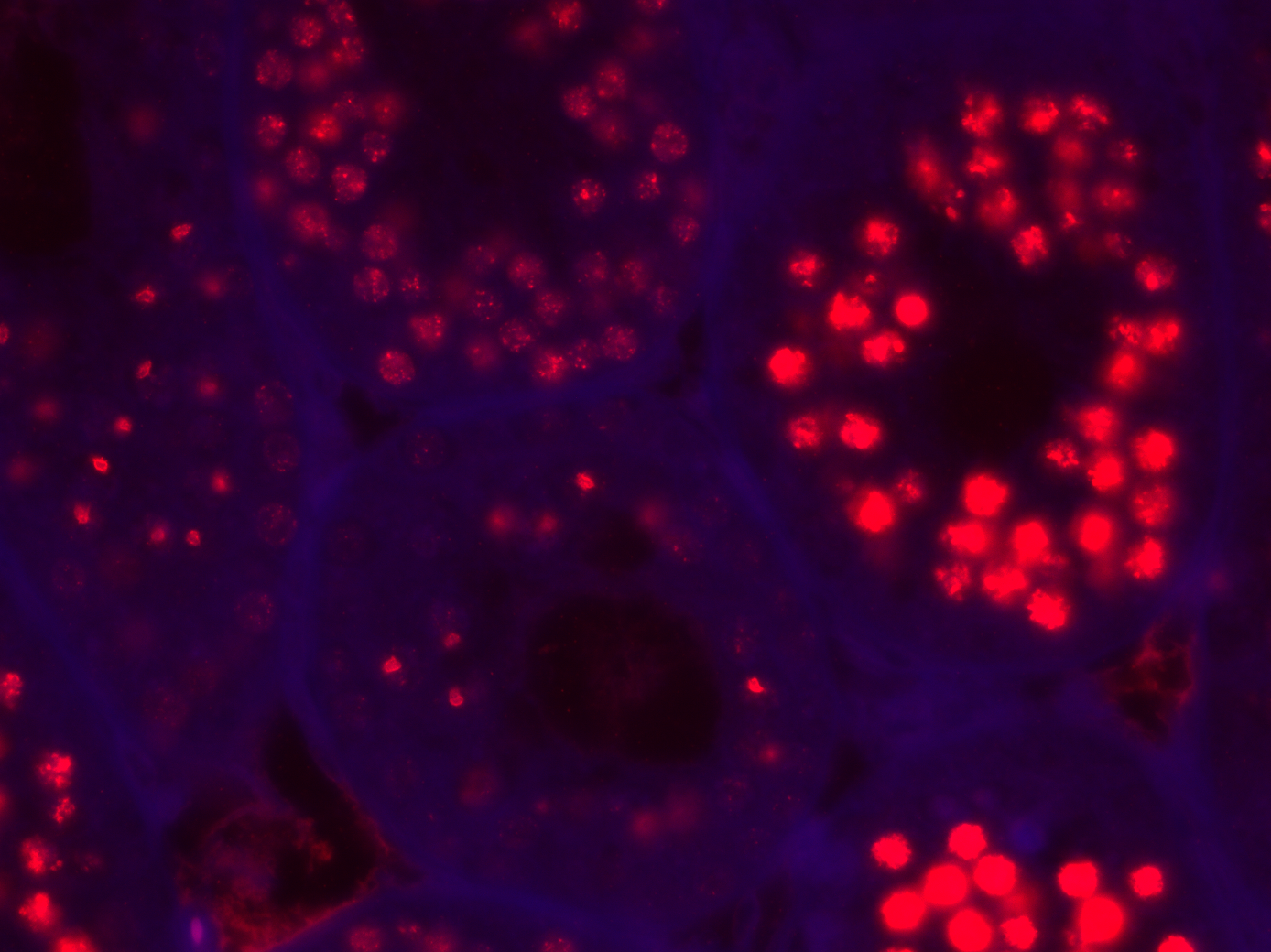

Supplement: Supplementary file 11 — Source data Fig. 3 [file 44318_2024_344_MOESM11_ESM.zip › Figure 3/Fig 3A/Fig 3B wild type.tif]

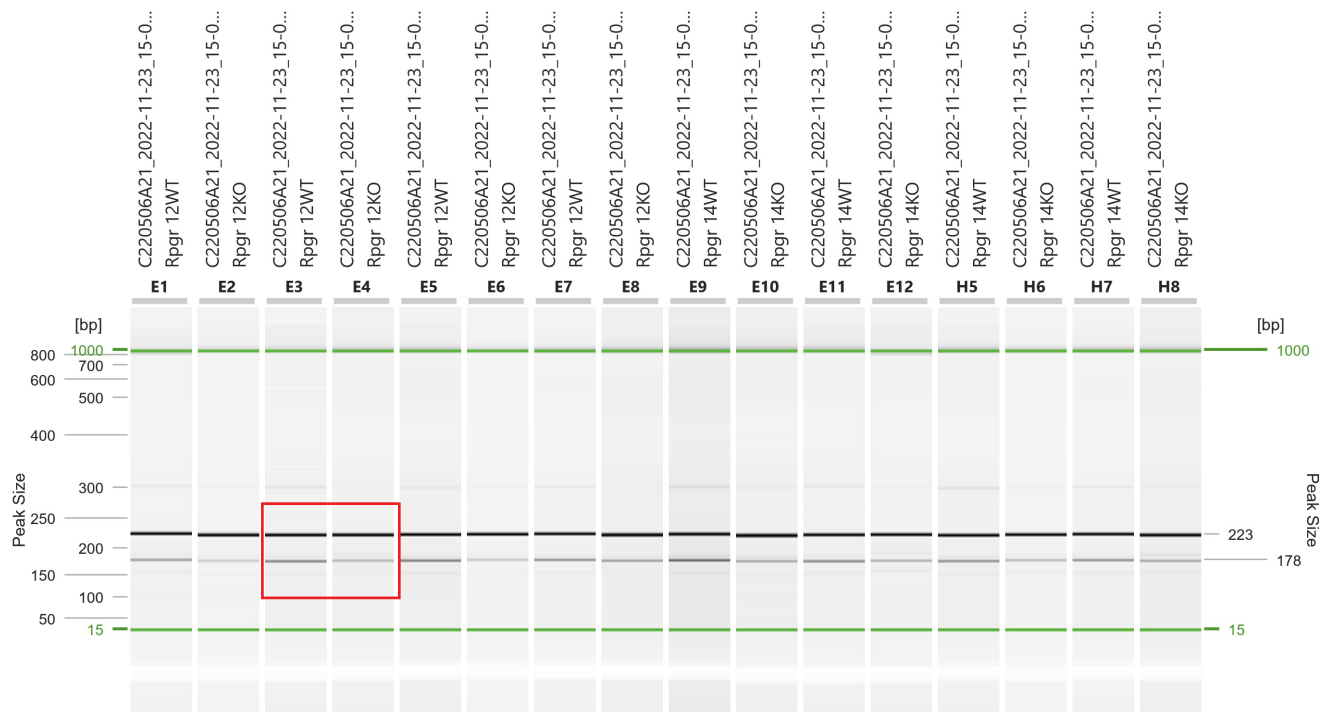

Supplement: Supplementary file 12 — Source data Fig. 4 [file 44318_2024_344_MOESM12_ESM.zip › Figure 4/Rpgr.pdf]

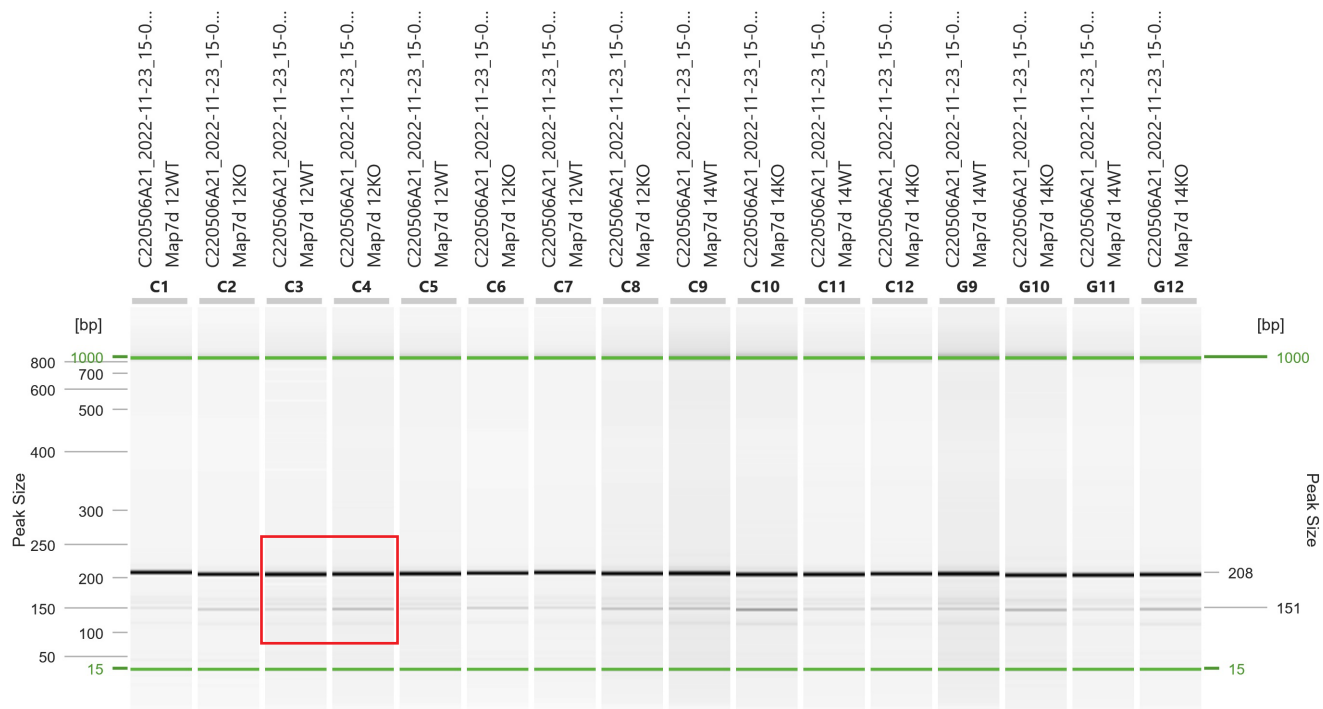

Supplement: Supplementary file 12 — Source data Fig. 4 [file 44318_2024_344_MOESM12_ESM.zip › Figure 4/map7d2.pdf]

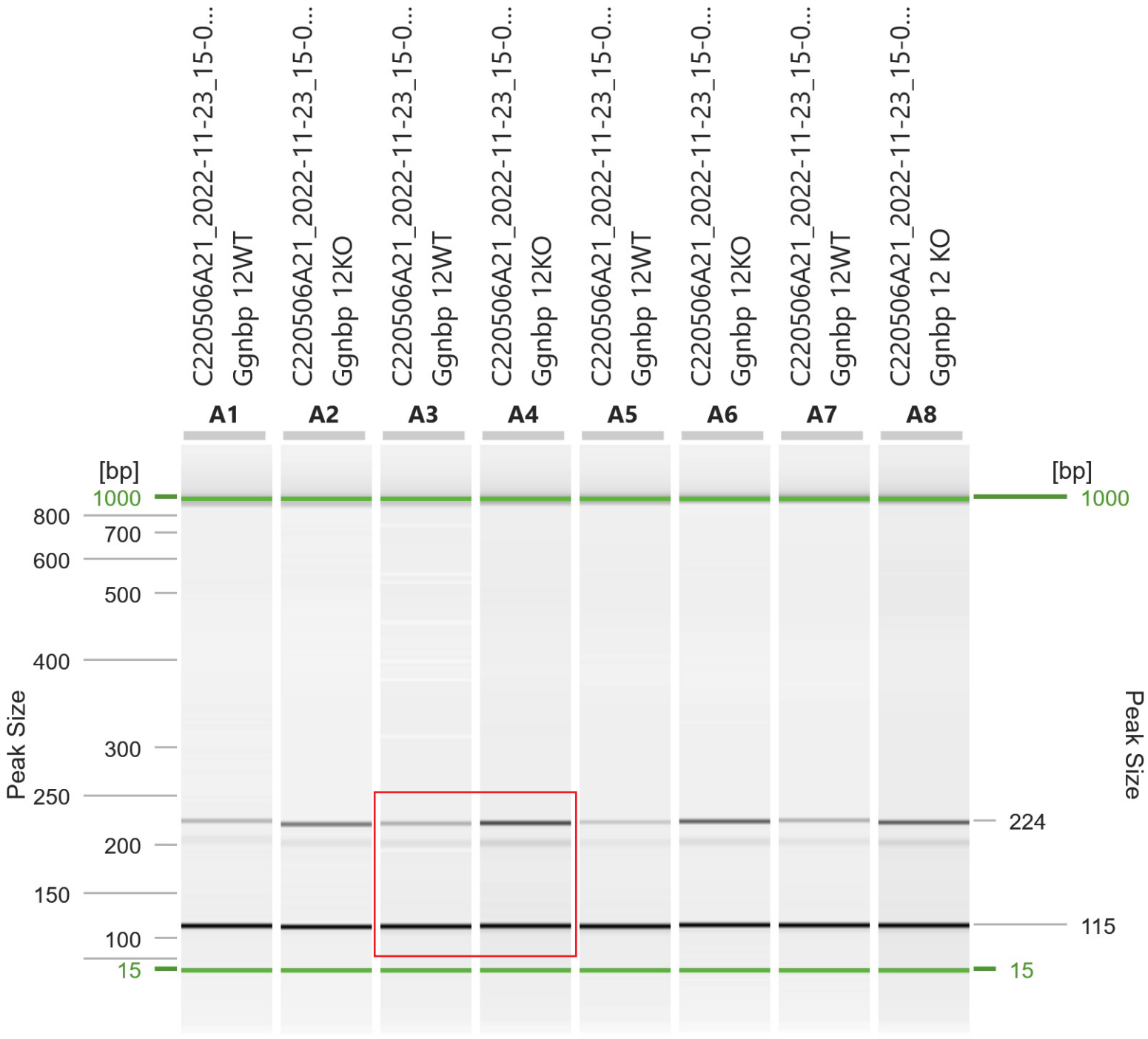

Supplement: Supplementary file 12 — Source data Fig. 4 [file 44318_2024_344_MOESM12_ESM.zip › Figure 4/Ggnbp2.pdf]

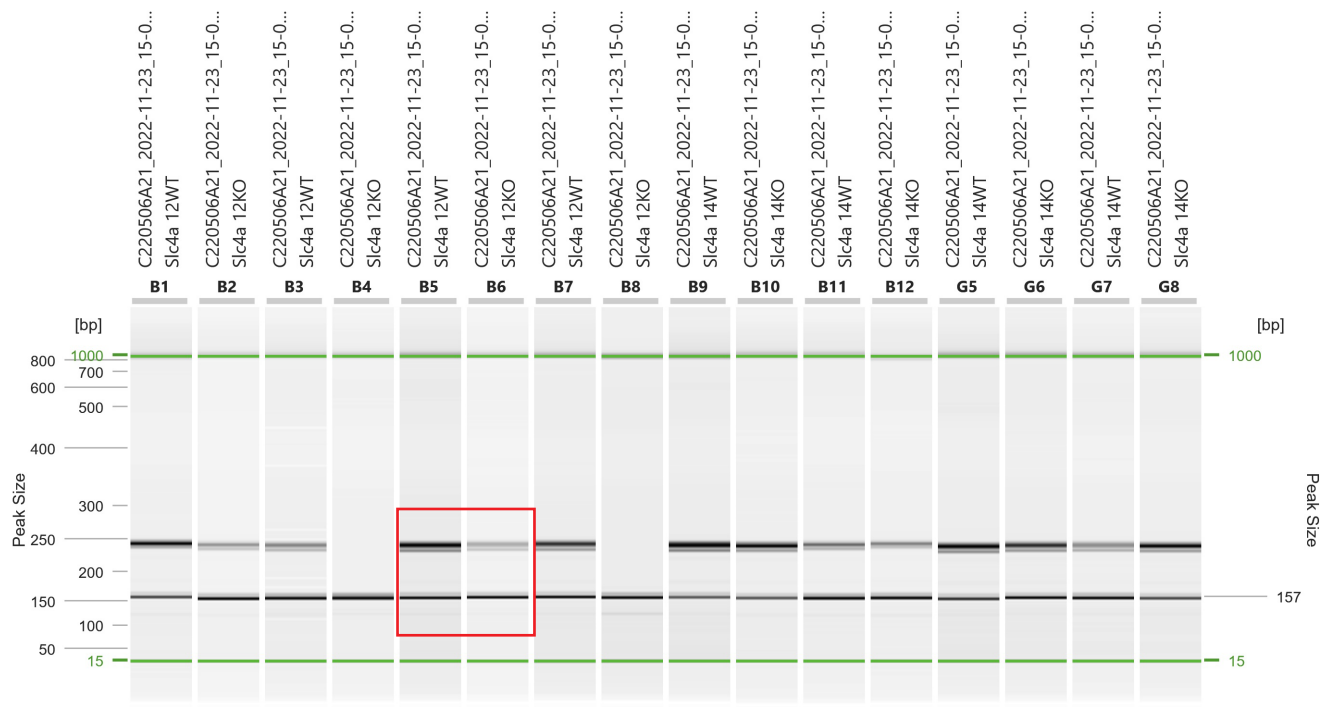

Supplement: Supplementary file 12 — Source data Fig. 4 [file 44318_2024_344_MOESM12_ESM.zip › Figure 4/Slc4a1ap.pdf]

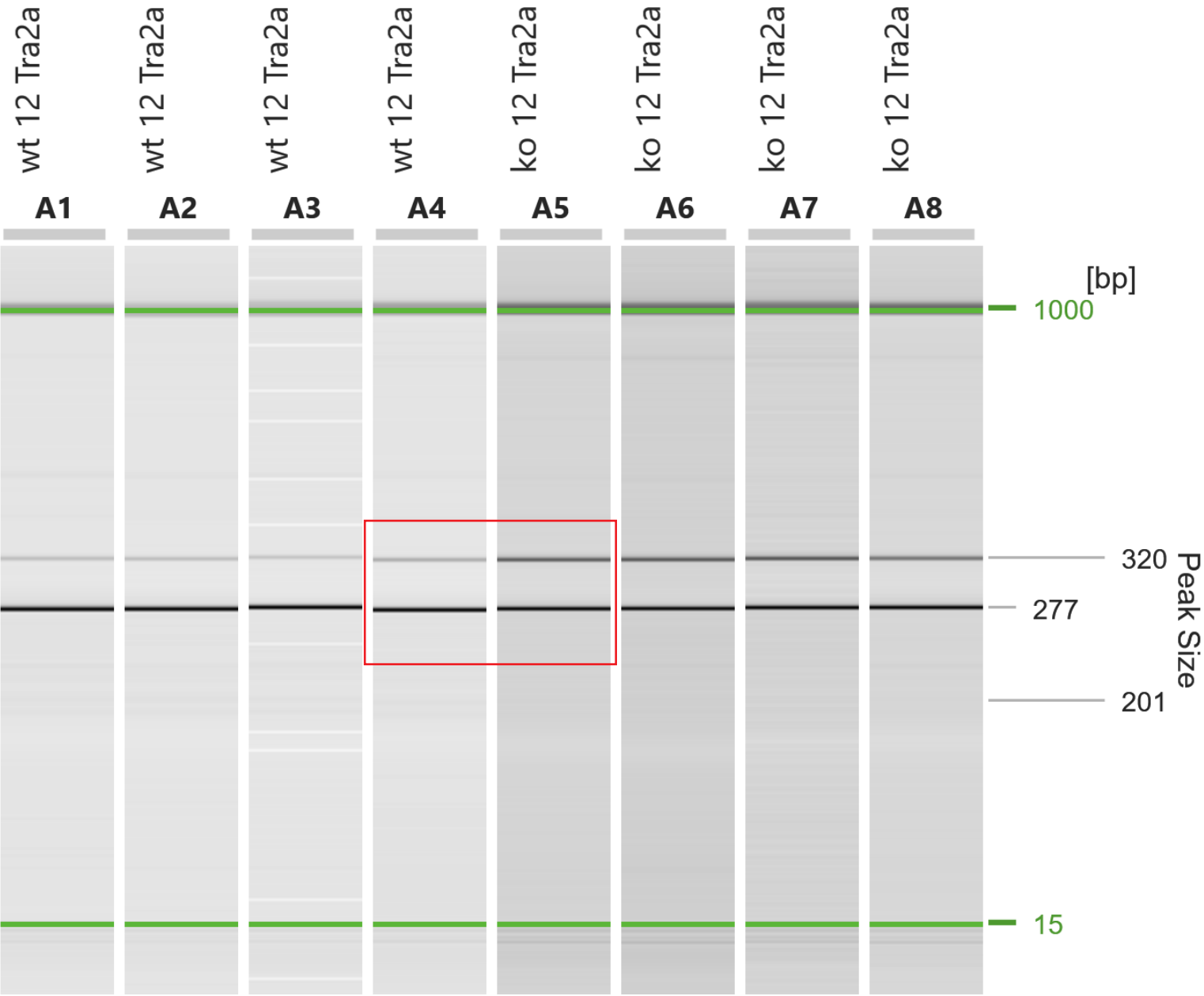

Supplement: Supplementary file 12 — Source data Fig. 4 [file 44318_2024_344_MOESM12_ESM.zip › Figure 4/Tra2a.pdf]

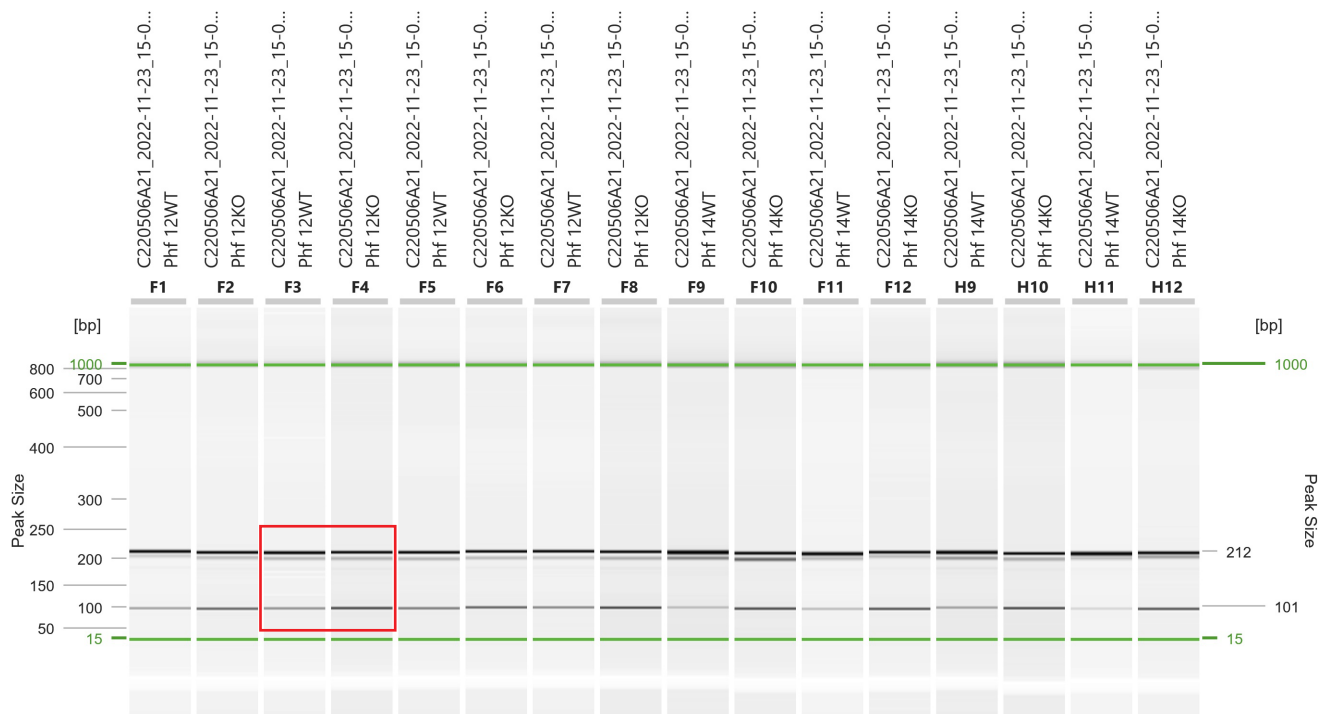

Supplement: Supplementary file 12 — Source data Fig. 4 [file 44318_2024_344_MOESM12_ESM.zip › Figure 4/PHF7 Fig3.pdf]

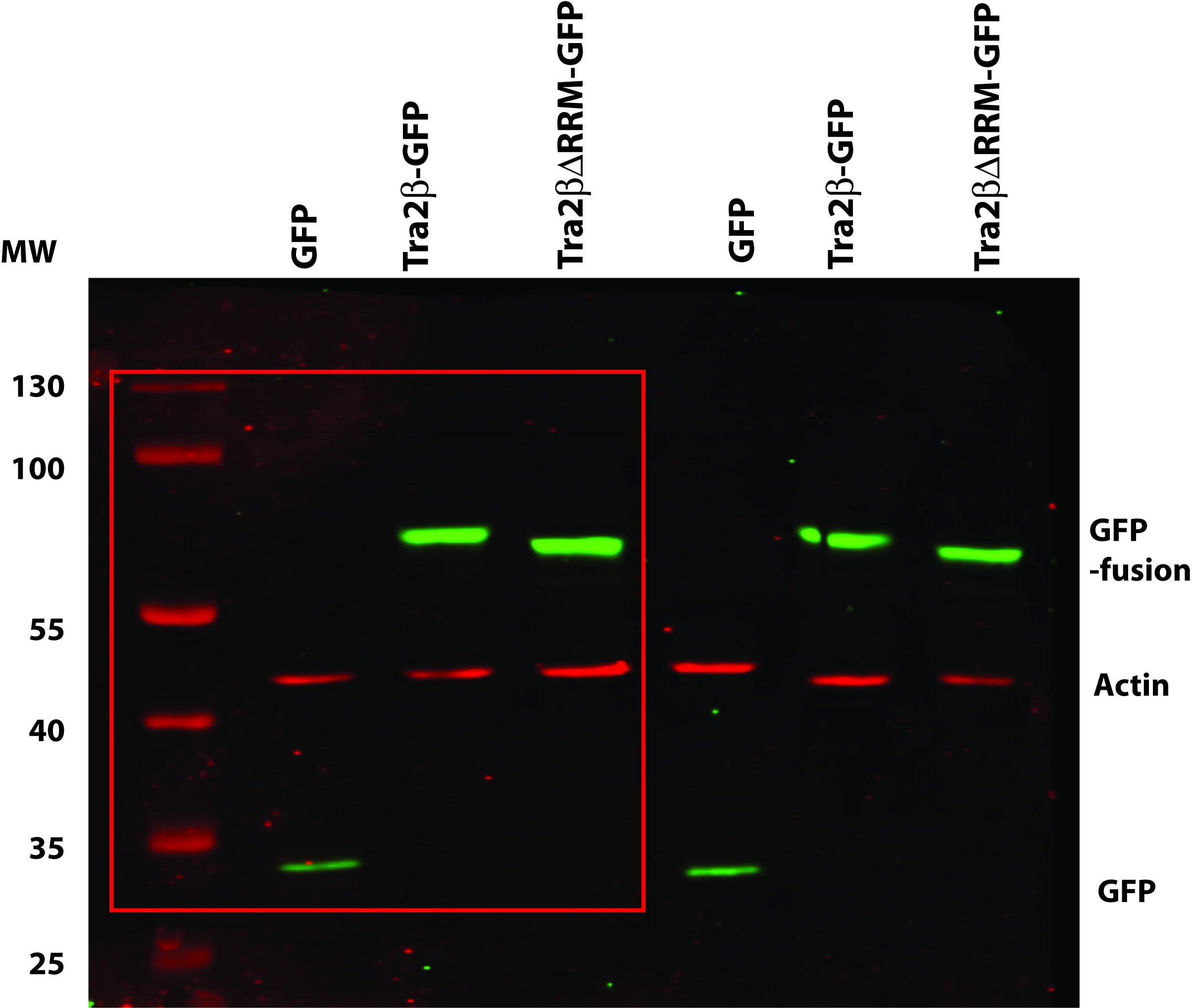

Supplement: Supplementary file 13 — Source data Fig. 5 [file 44318_2024_344_MOESM13_ESM.zip › Figure 5/Figure 5F original image.tif]

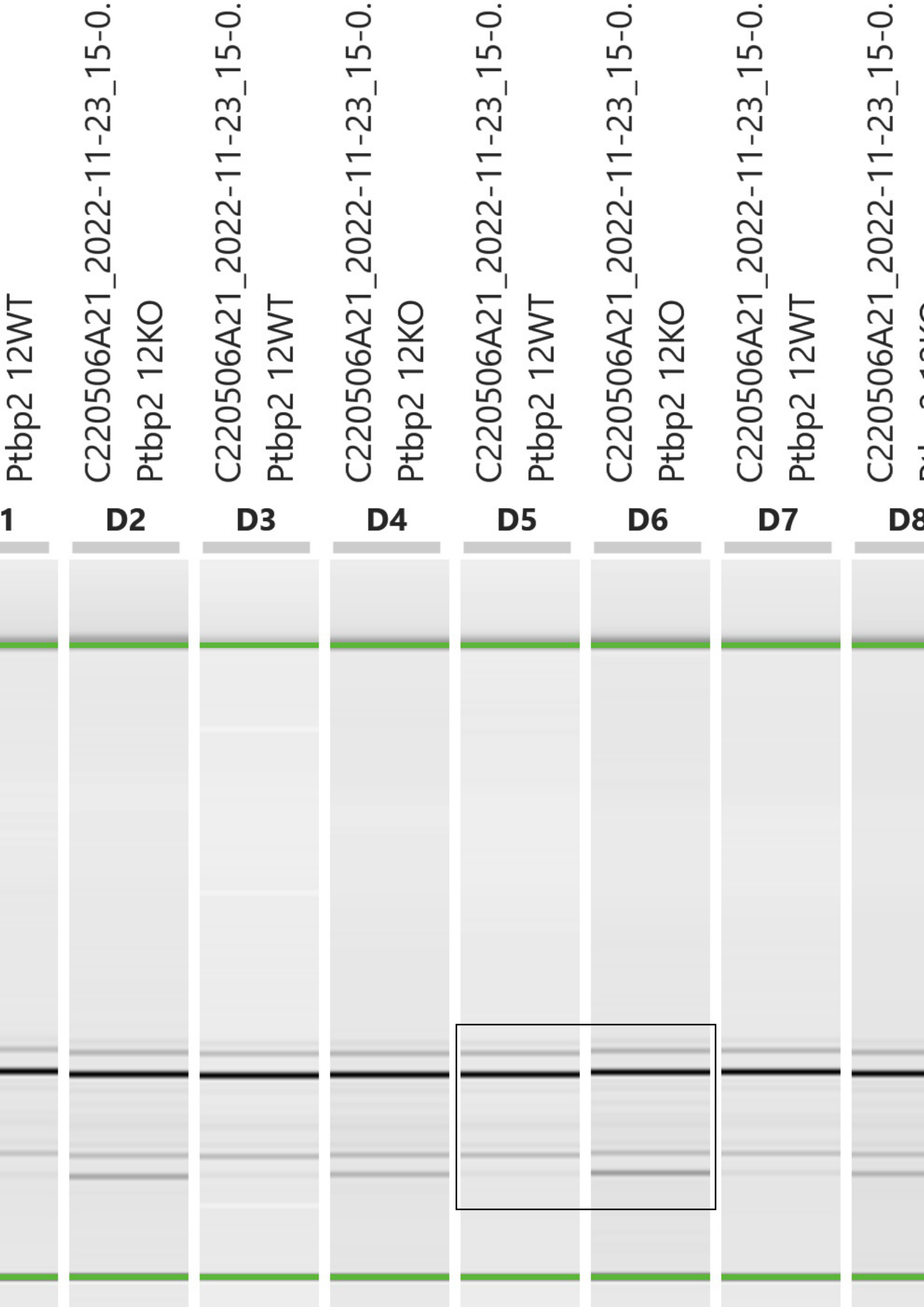

Supplement: Supplementary file 13 — Source data Fig. 5 [file 44318_2024_344_MOESM13_ESM.zip › Figure 5/5B original electrophoretogram.pdf]

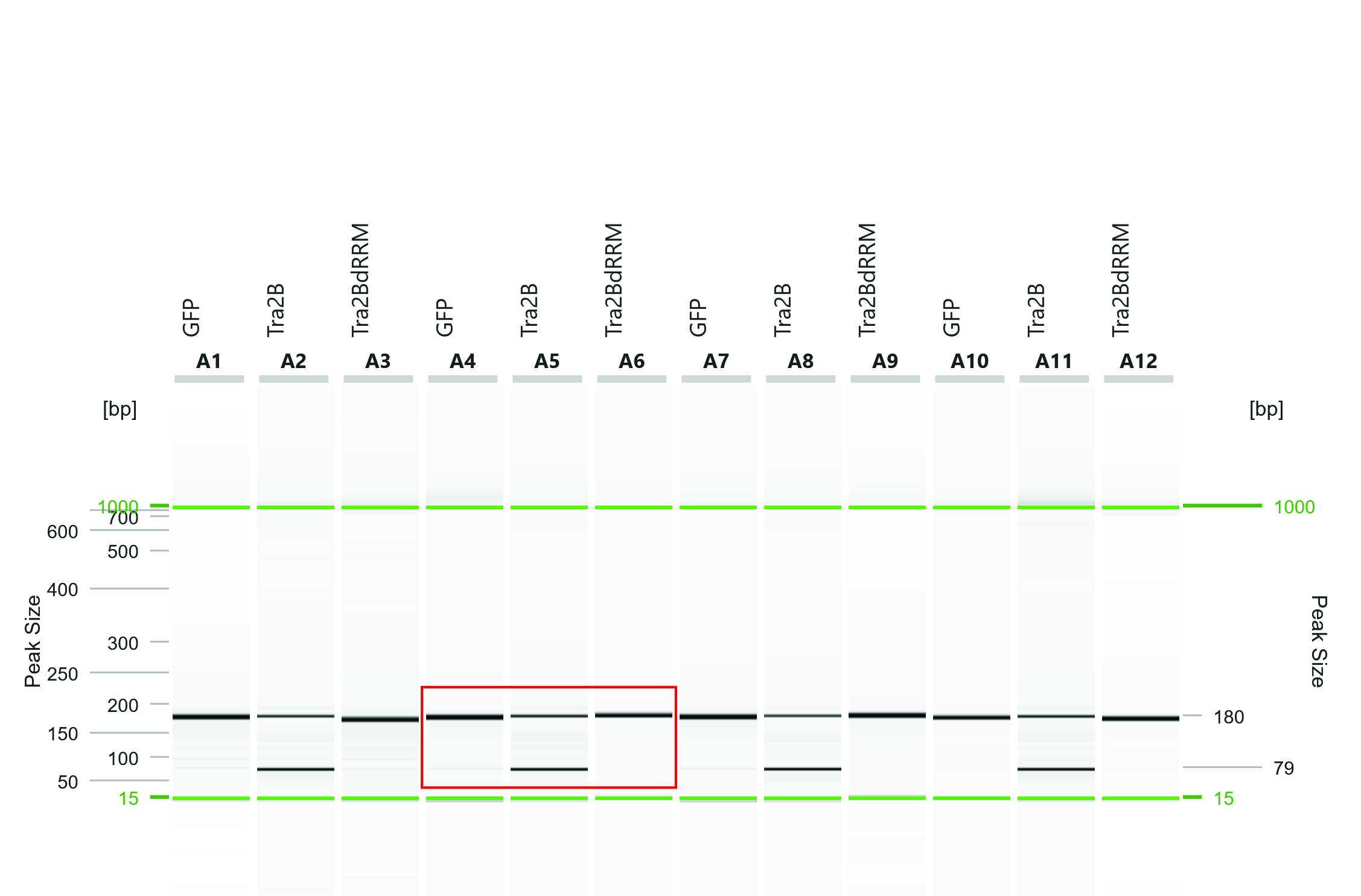

Supplement: Supplementary file 13 — Source data Fig. 5 [file 44318_2024_344_MOESM13_ESM.zip › Figure 5/5E original image.tif]

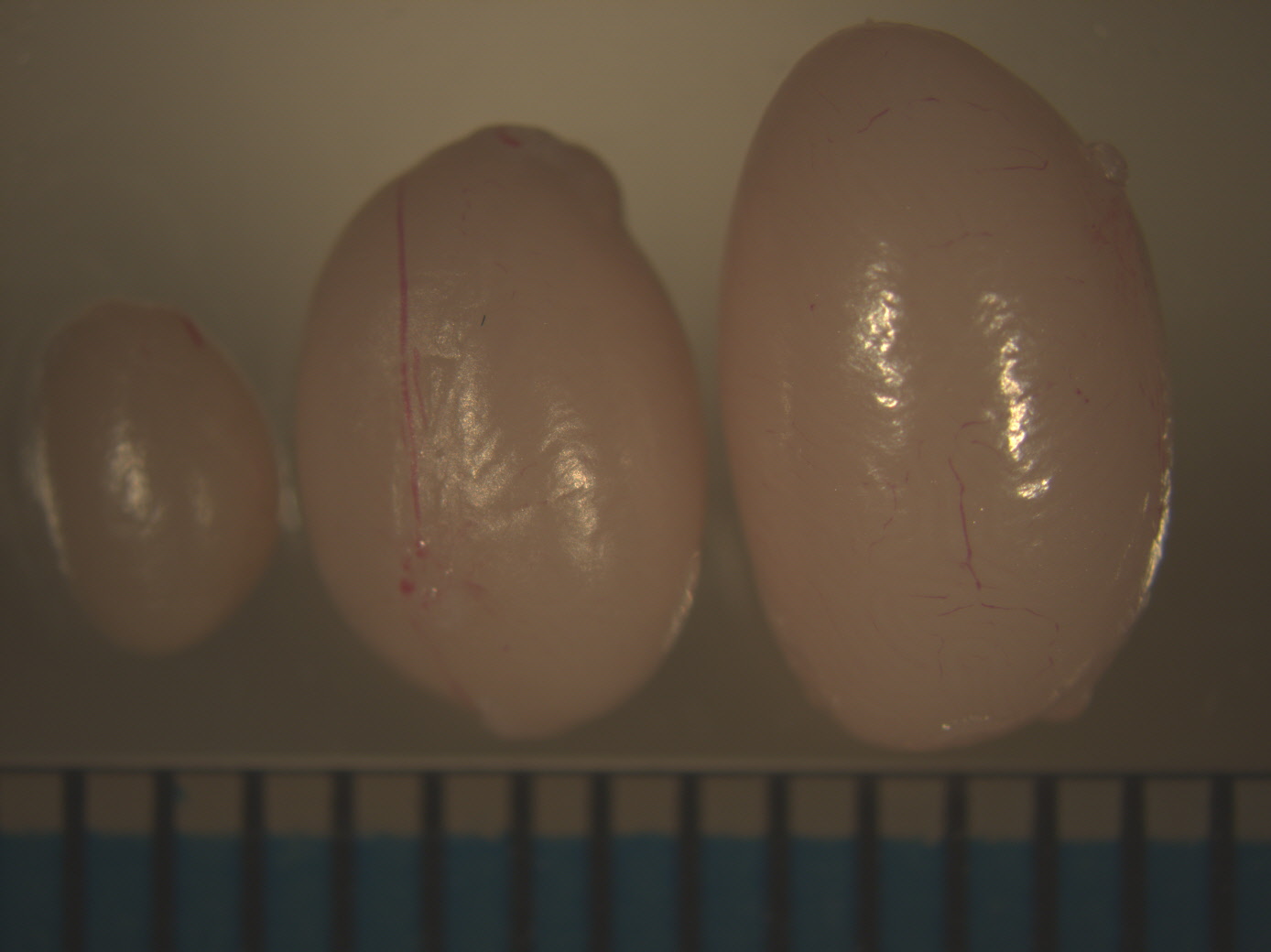

Supplement: Supplementary file 14 — Source data Fig. 6 [file 44318_2024_344_MOESM14_ESM.zip › Figure 6/Fig 6B.JPG]

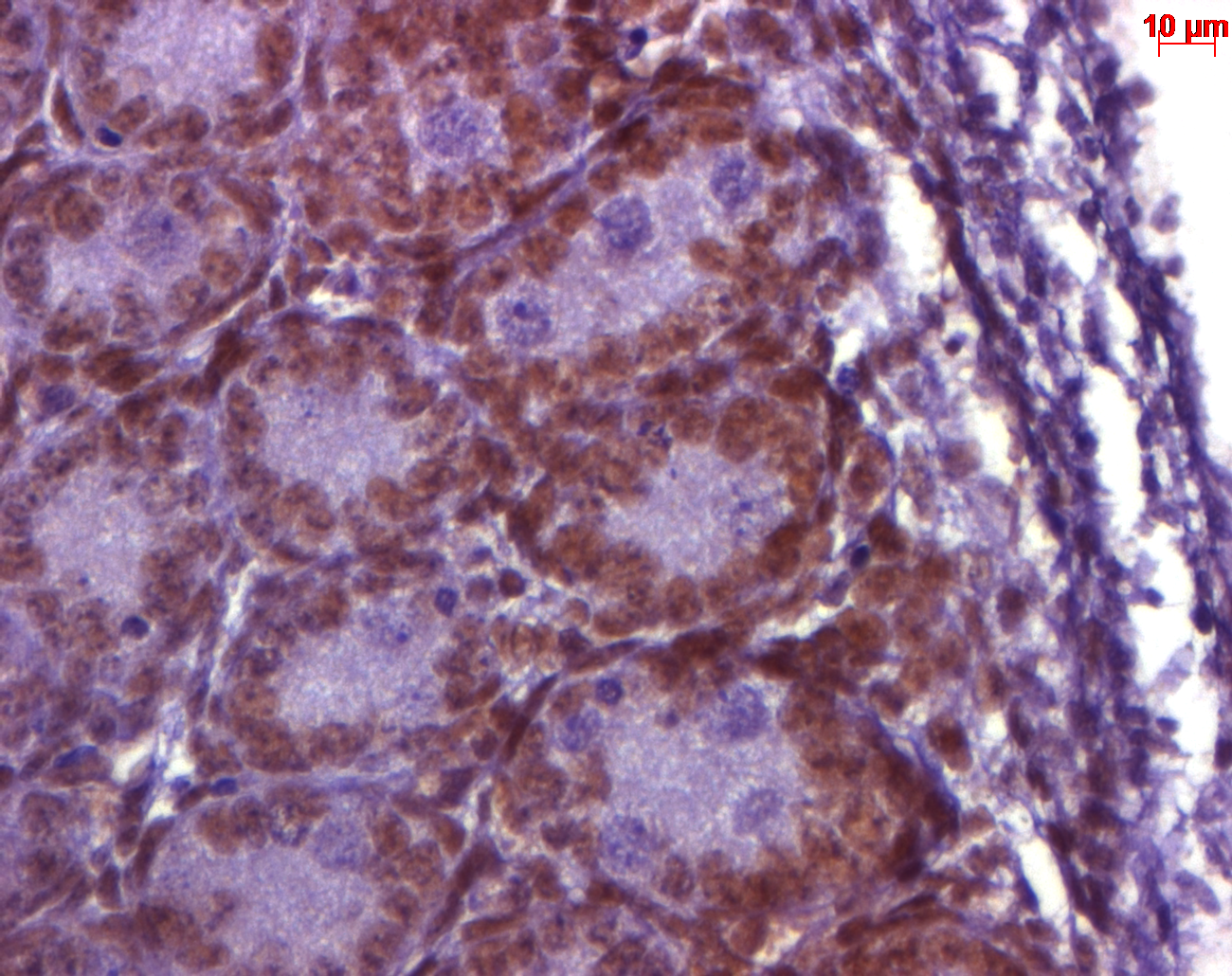

Supplement: Supplementary file 14 — Source data Fig. 6 [file 44318_2024_344_MOESM14_ESM.zip › Figure 6/Figure 6G panels/P1 Tra2b-cKO stained for Tra2b protein.TIF]

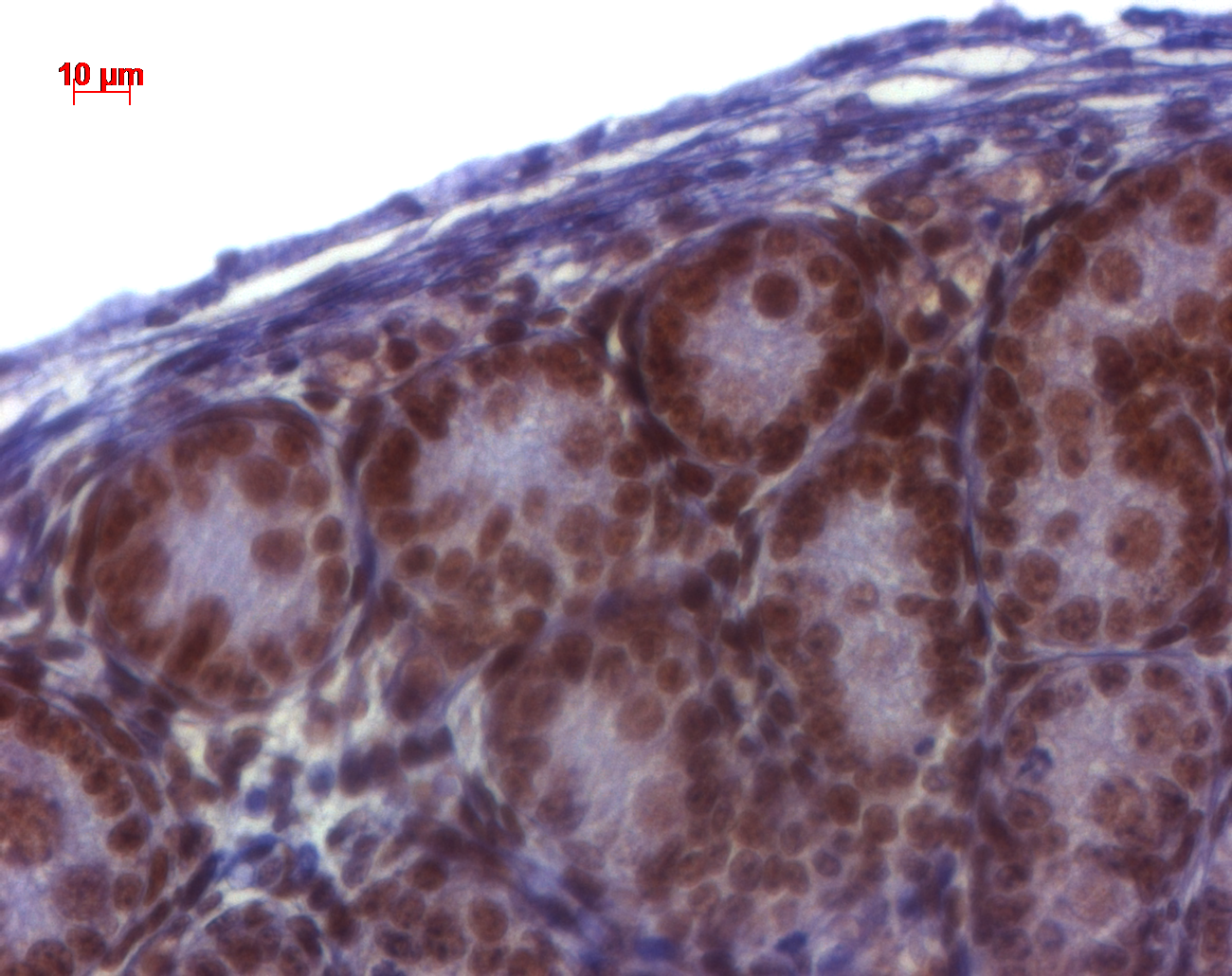

Supplement: Supplementary file 14 — Source data Fig. 6 [file 44318_2024_344_MOESM14_ESM.zip › Figure 6/Figure 6G panels/P1 wild type testis stained for Tra2b protein.TIF]

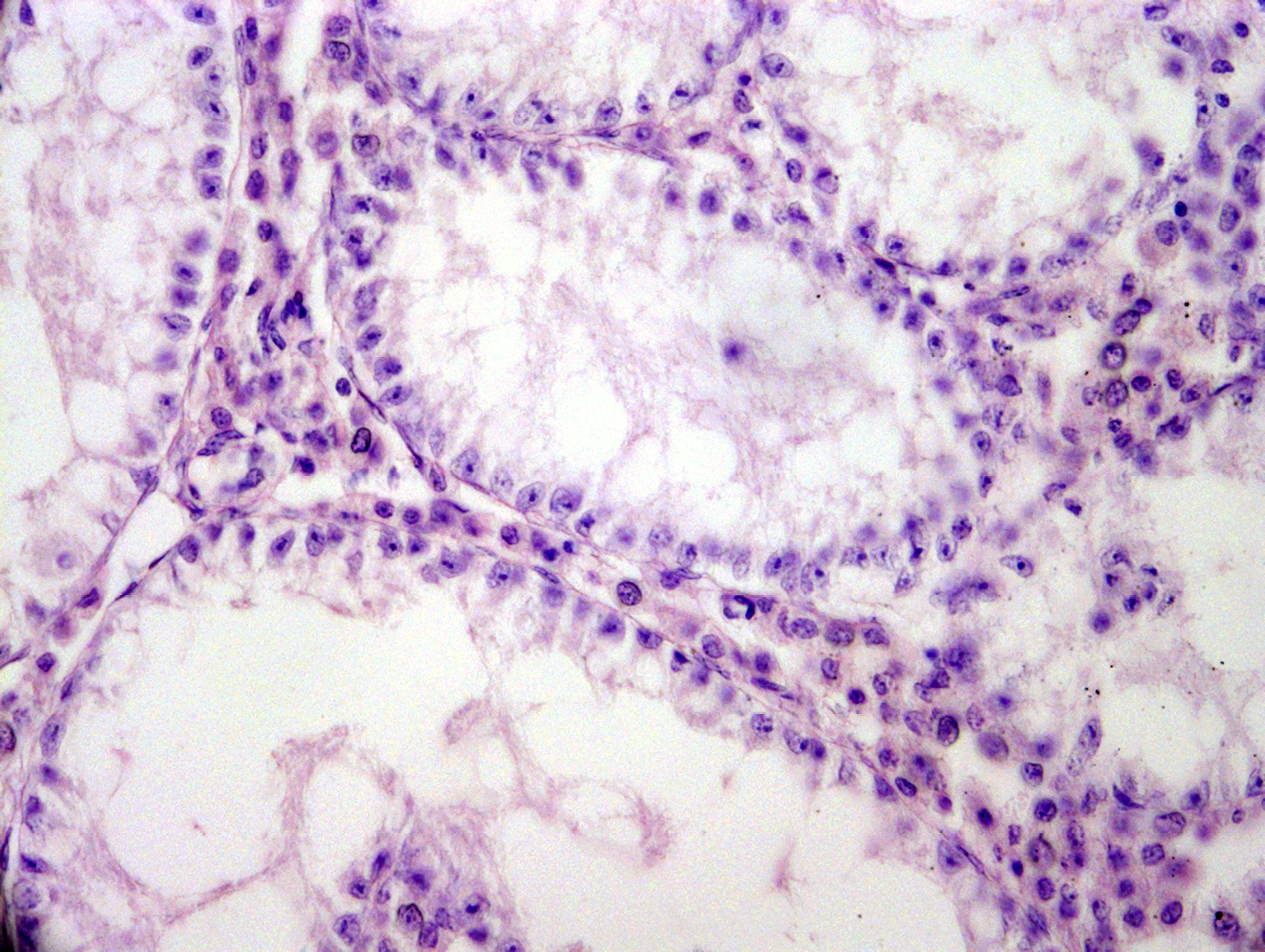

Supplement: Supplementary file 14 — Source data Fig. 6 [file 44318_2024_344_MOESM14_ESM.zip › Figure 6/Figure 6E panels/Fig 6E Tra2b-cko.tif]

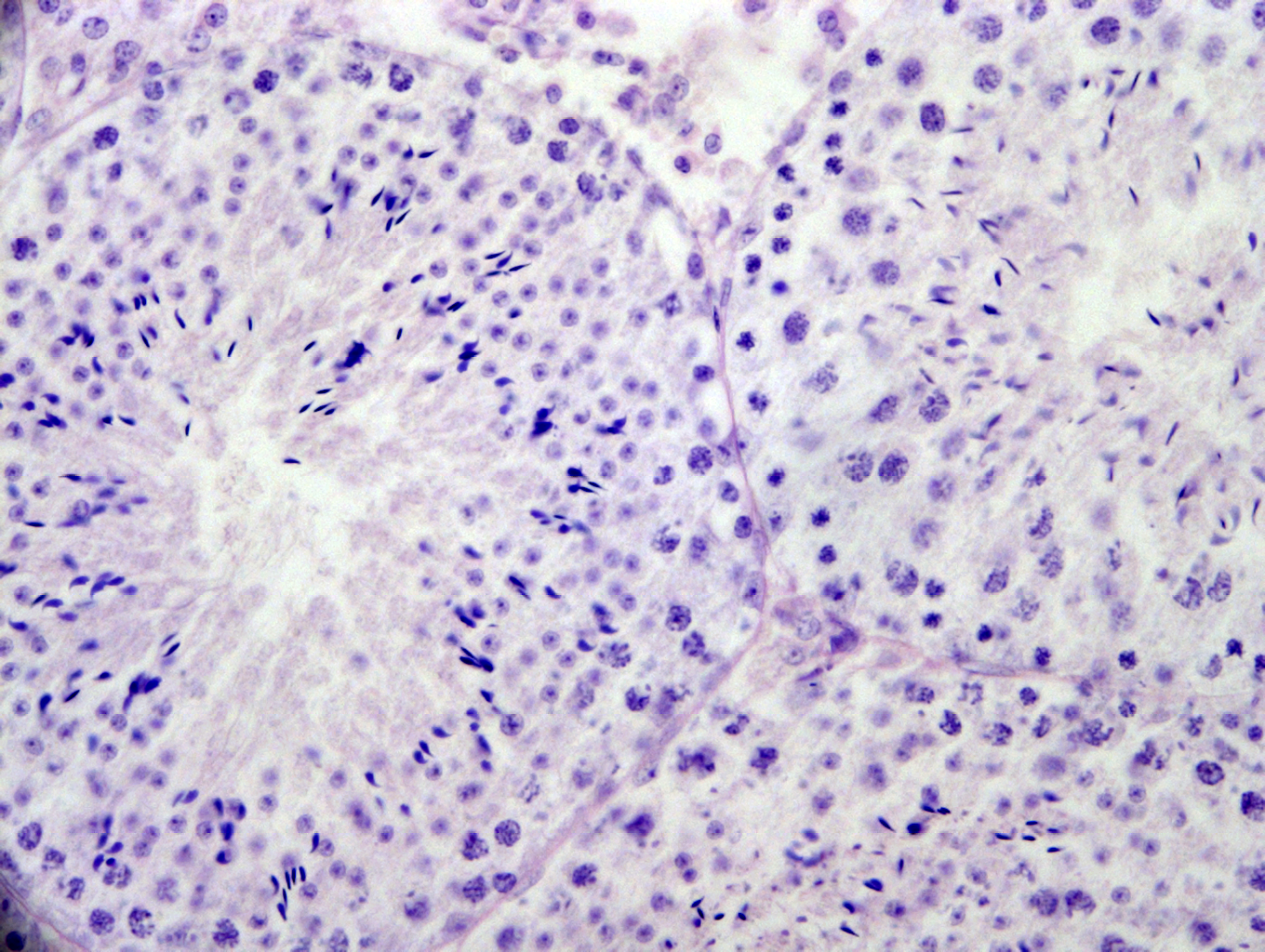

Supplement: Supplementary file 14 — Source data Fig. 6 [file 44318_2024_344_MOESM14_ESM.zip › Figure 6/Figure 6E panels/6E wild type.tif]

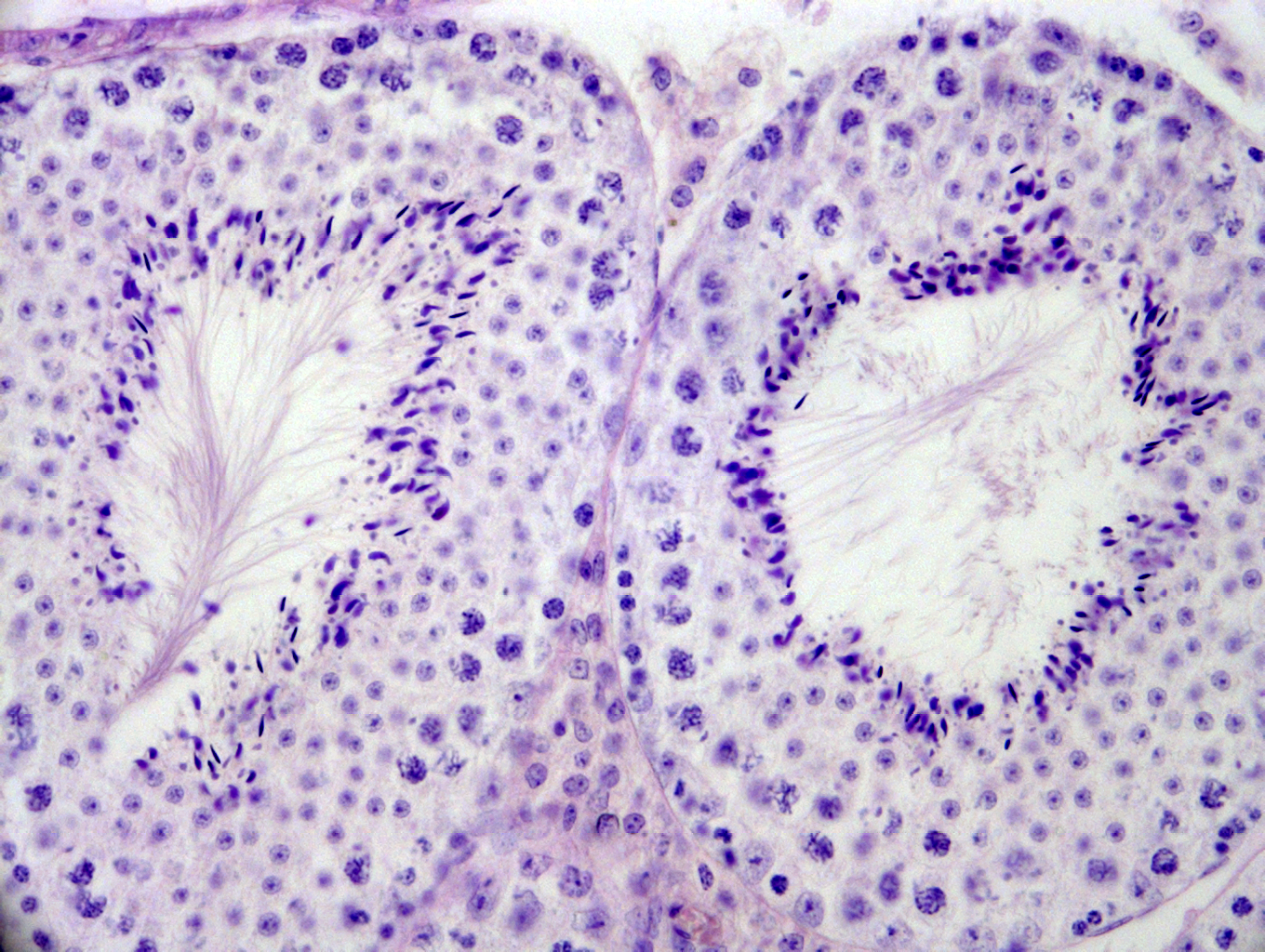

Supplement: Supplementary file 14 — Source data Fig. 6 [file 44318_2024_344_MOESM14_ESM.zip › Figure 6/Figure 6E panels/Fig 6E Tra2b-het.tif]

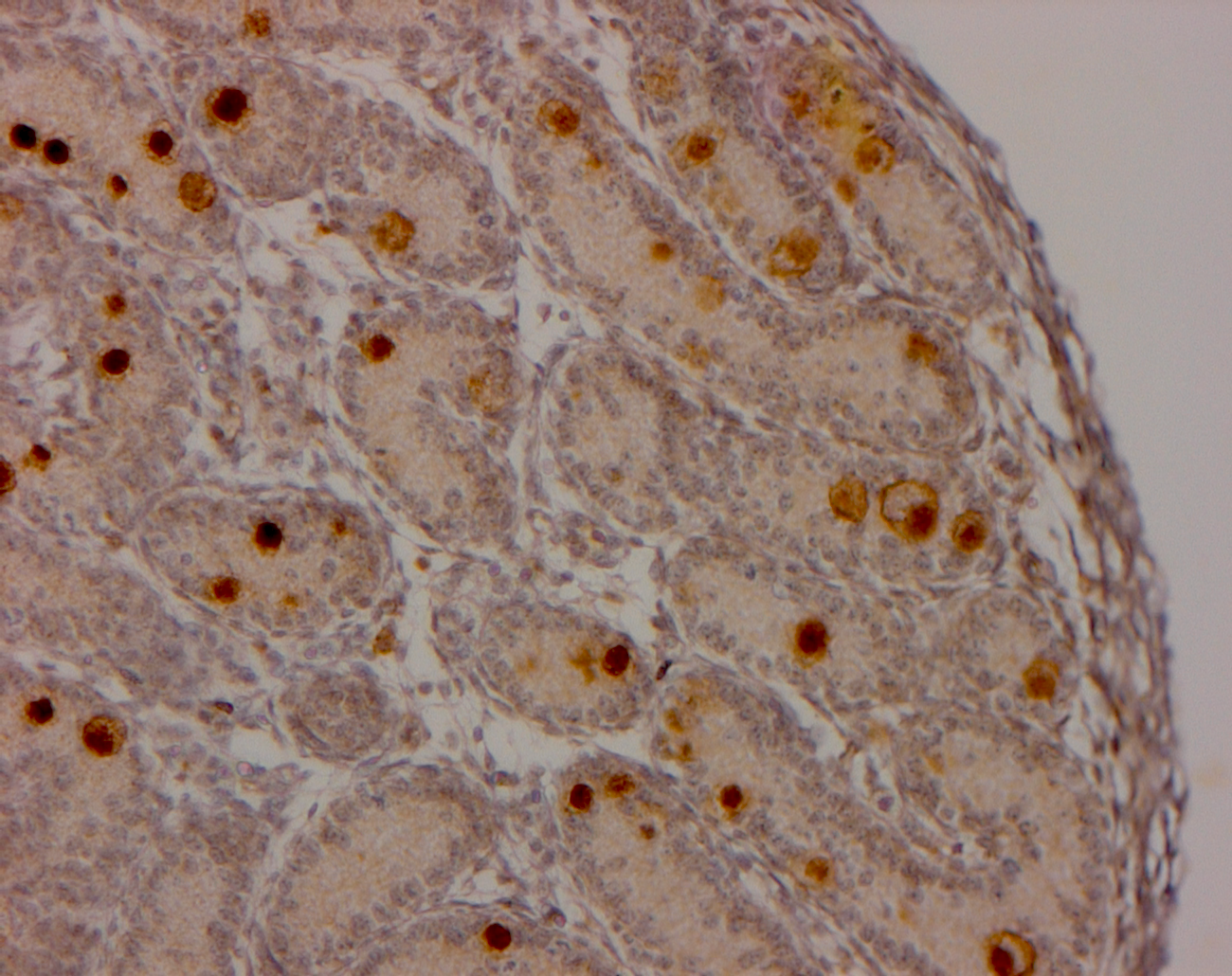

Supplement: Supplementary file 14 — Source data Fig. 6 [file 44318_2024_344_MOESM14_ESM.zip › Figure 6/Figure 6F/P3 Tra2b-cKO testis stained for RBMY.tif]

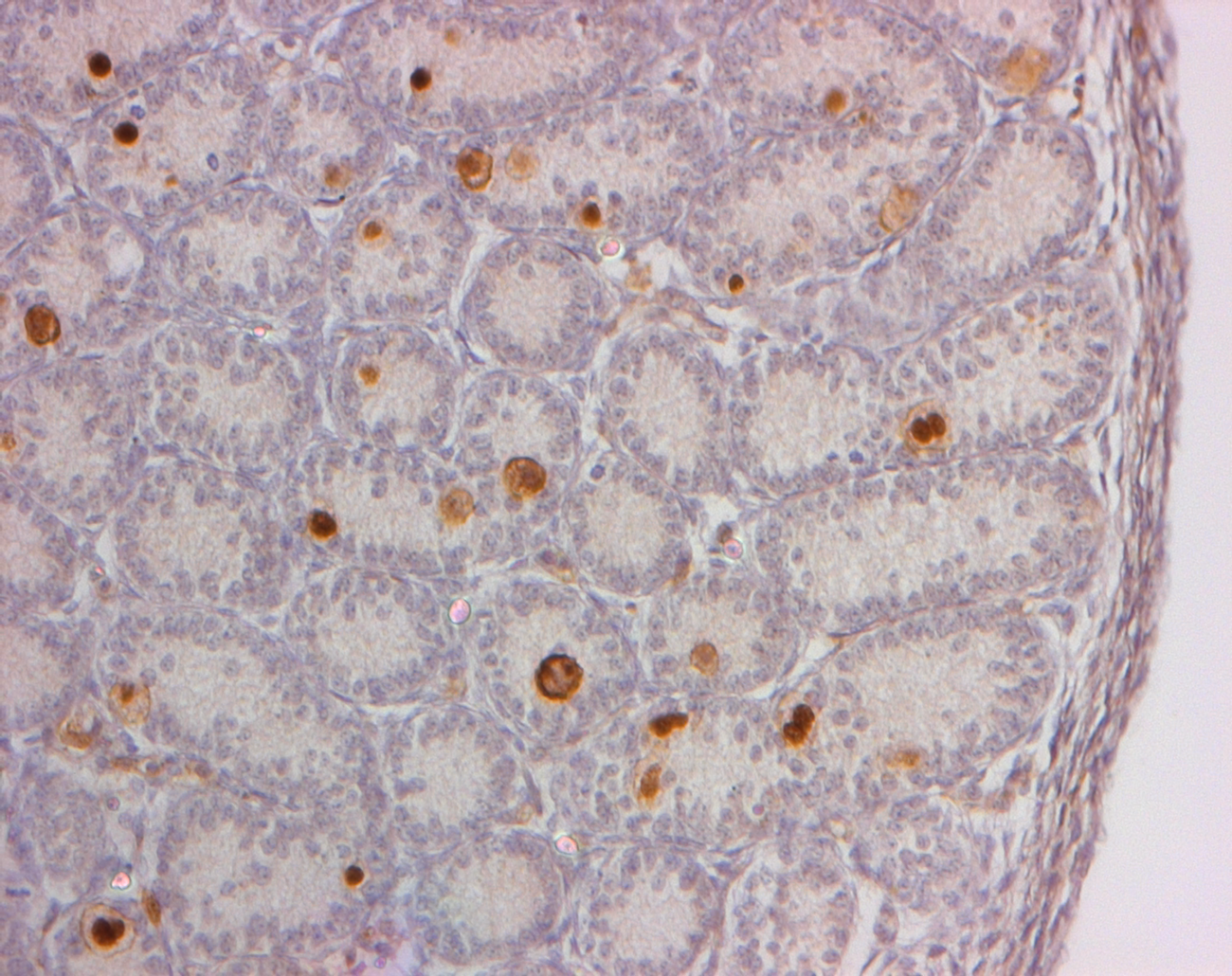

Supplement: Supplementary file 14 — Source data Fig. 6 [file 44318_2024_344_MOESM14_ESM.zip › Figure 6/Figure 6F/P2 Tra2b-cKO testis stained for RBMY.tif]

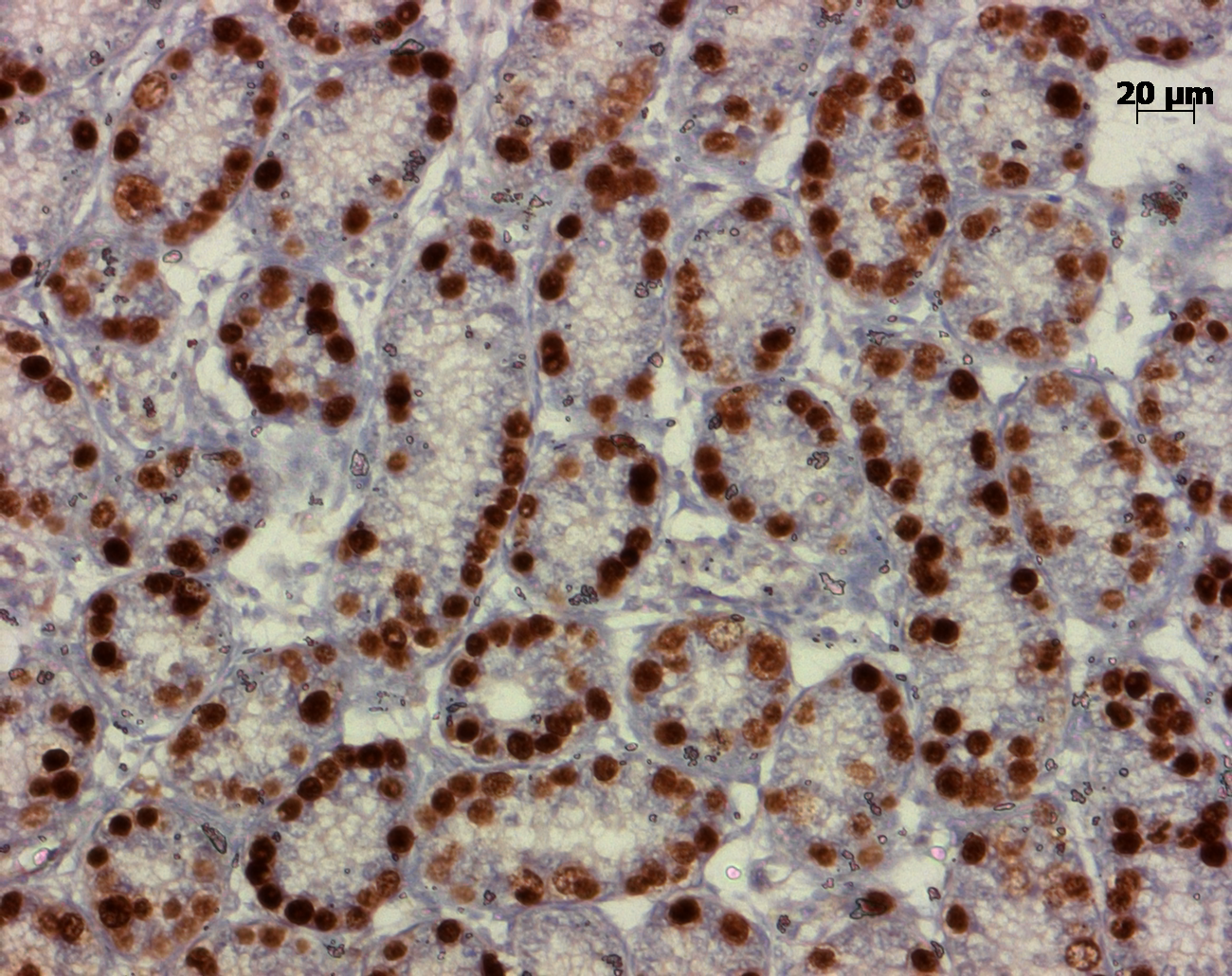

Supplement: Supplementary file 14 — Source data Fig. 6 [file 44318_2024_344_MOESM14_ESM.zip › Figure 6/Figure 6F/P3 Tra2b-het testis stained for RBMY.TIF]

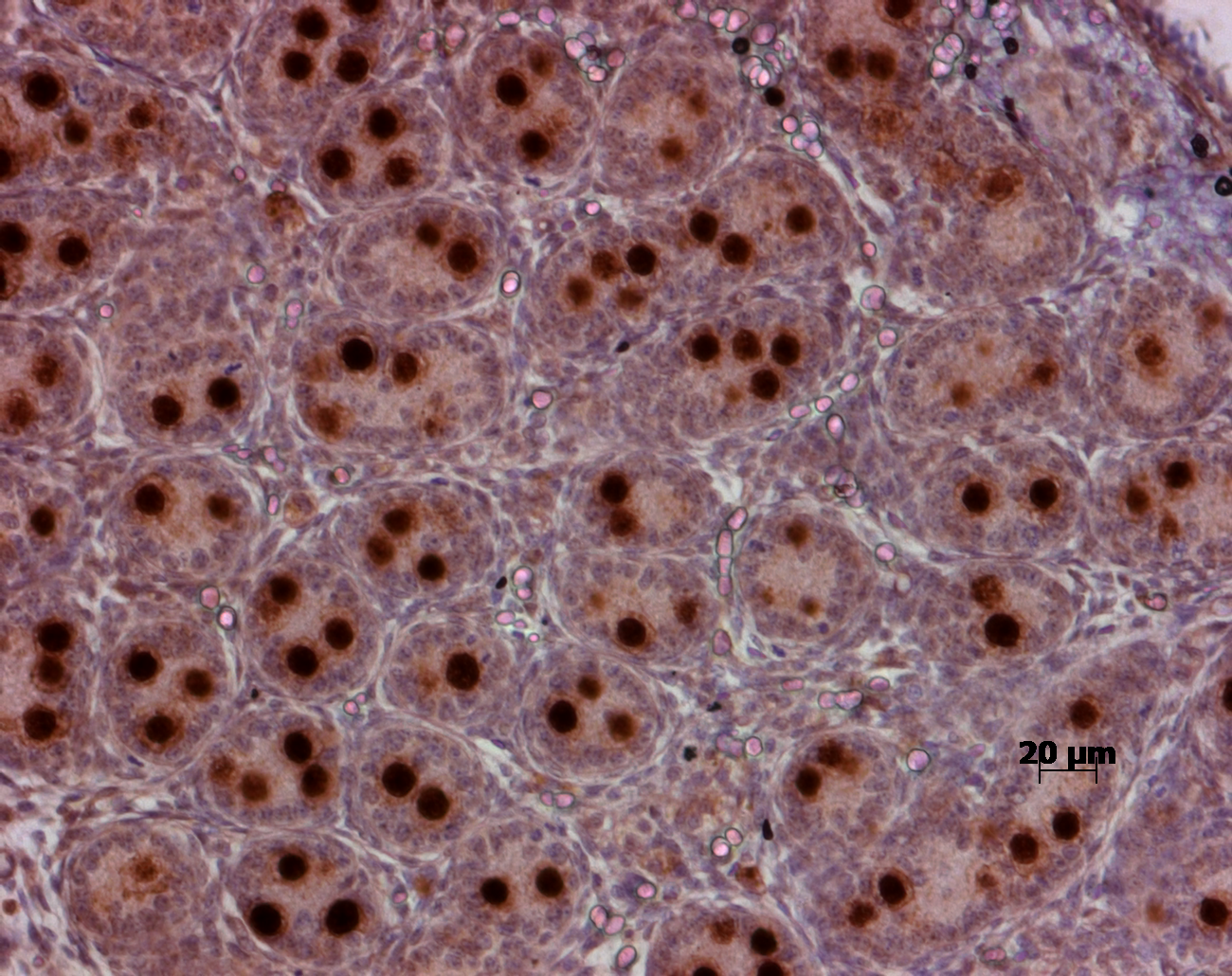

Supplement: Supplementary file 14 — Source data Fig. 6 [file 44318_2024_344_MOESM14_ESM.zip › Figure 6/Figure 6F/P1 wild type testis stained for RBMY.TIF]

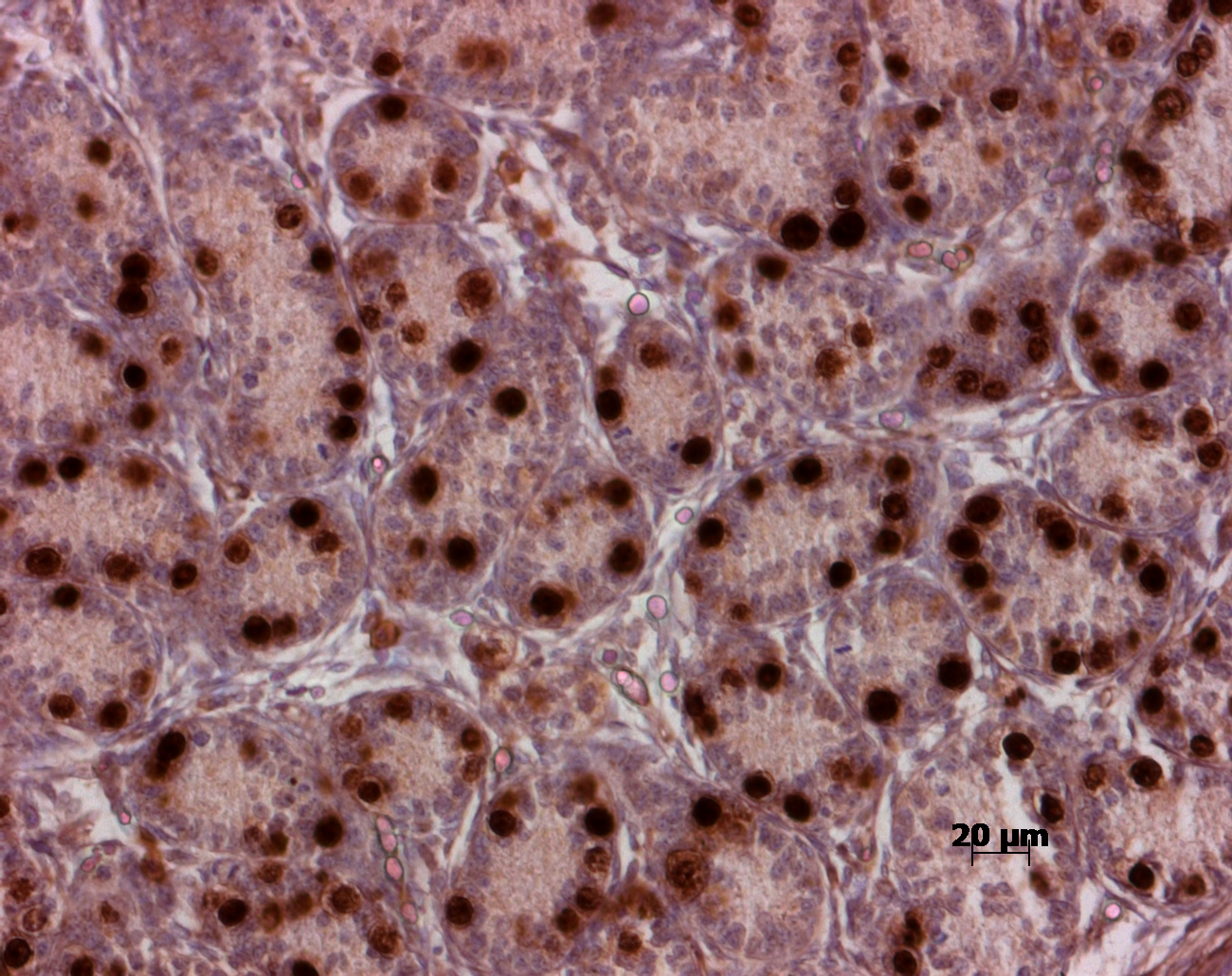

Supplement: Supplementary file 14 — Source data Fig. 6 [file 44318_2024_344_MOESM14_ESM.zip › Figure 6/Figure 6F/P2 wild type testis stained for RBMY.TIF]

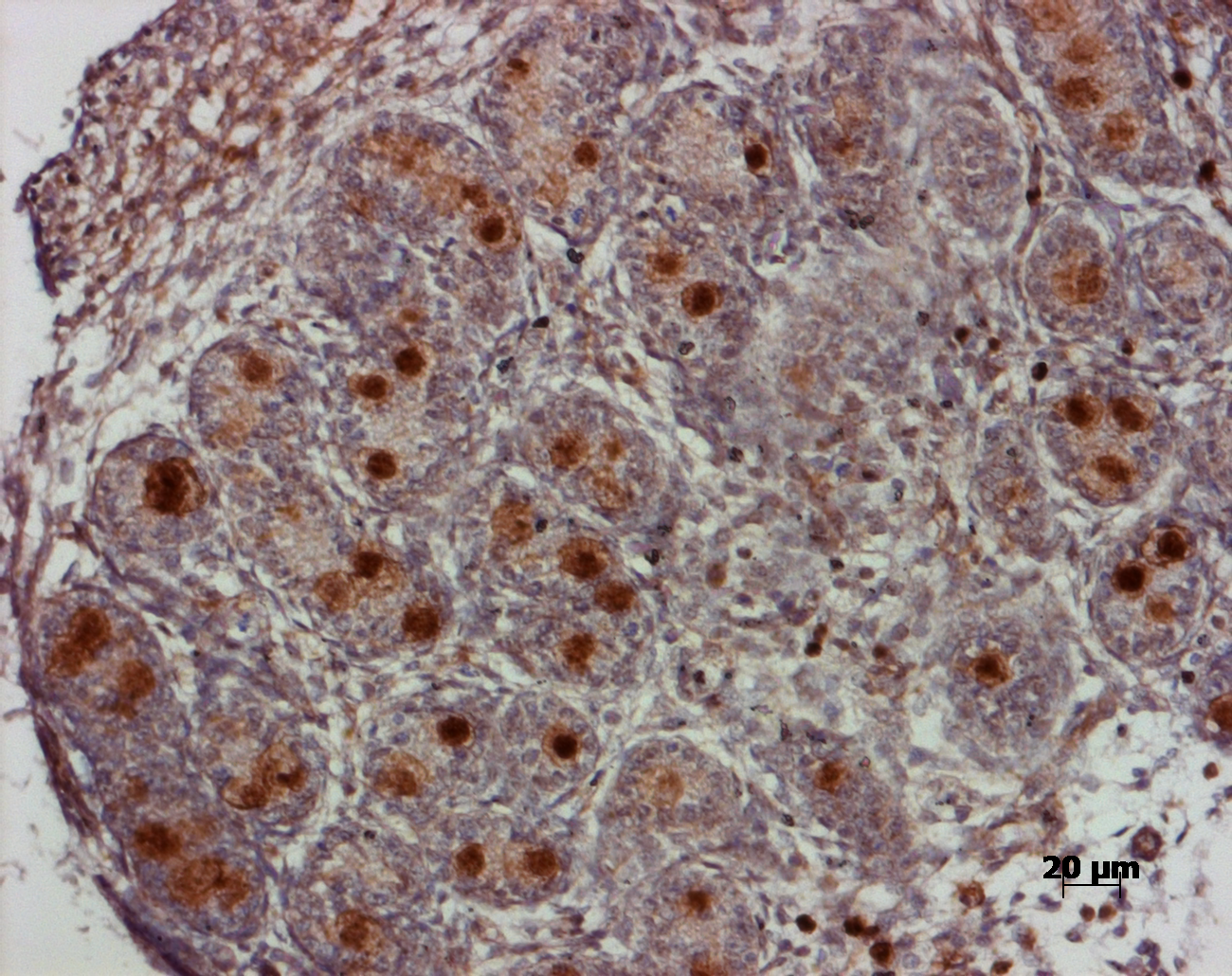

Supplement: Supplementary file 14 — Source data Fig. 6 [file 44318_2024_344_MOESM14_ESM.zip › Figure 6/Figure 6F/PO Tra2b-cKO stained for RBMY.TIF]

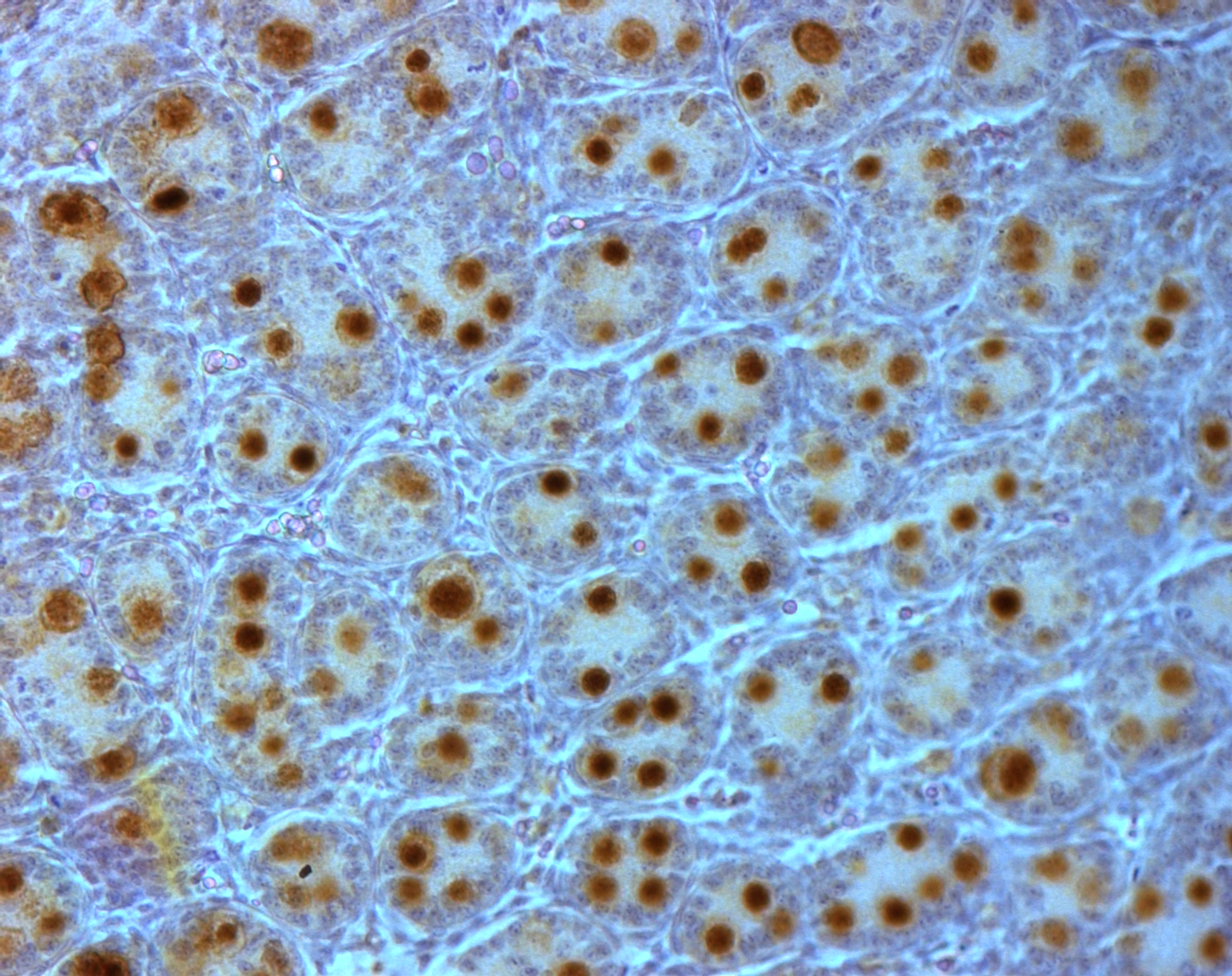

Supplement: Supplementary file 14 — Source data Fig. 6 [file 44318_2024_344_MOESM14_ESM.zip › Figure 6/Figure 6F/P1 Tra2b-cKO testis stained for RBMY.tif]

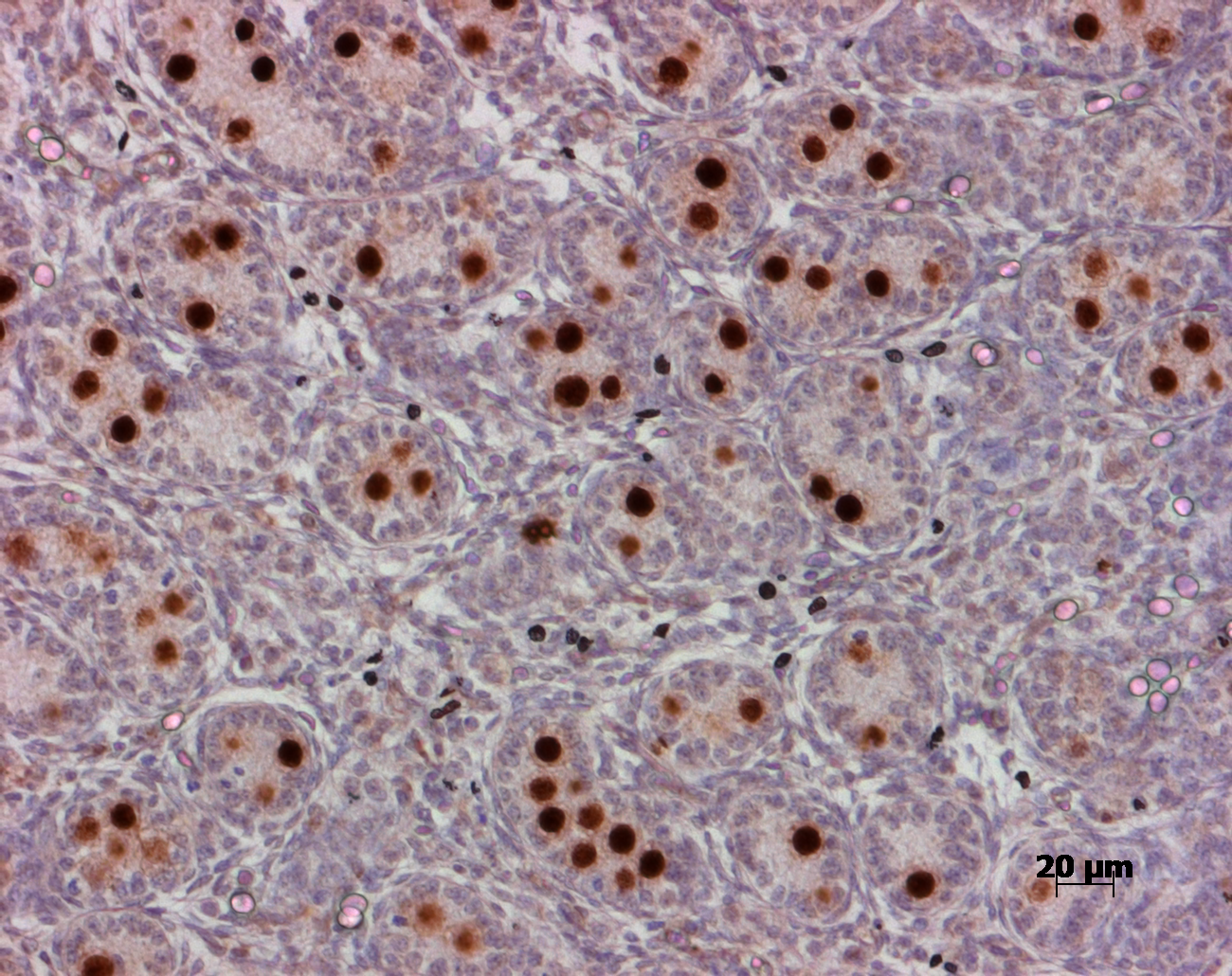

Supplement: Supplementary file 14 — Source data Fig. 6 [file 44318_2024_344_MOESM14_ESM.zip › Figure 6/Figure 6F/PO wild type testis stained for RBMY.TIF]
