# Supplementary material for: An ultra-conserved poison exon in the Tra2b gene encoding a splicing activator is essential for male fertility and meiotic cell division
Source: EMBO J. 2025 Jan 2;44(3):877–902. doi: 10.1038/s44318-024-00344-6 (PMC11791180; doi:10.1038/s44318-024-00344-6)
Supplement: Supplementary file 15 — Expanded View Figures [file 44318_2024_344_MOESM15_ESM.pdf]

Expanded View Figures

Figure EV1. Generation of mice with germ cell-specific deletion of the *Tra2b* PE.

(**A**) The *Tra2b* PE was flanked with *LoxP* sites. The blue primers bind only to the WT allele as the forward primer sits in the region that is removed and replaced by the insertion of the 5′ *LoxP* site. Thus, they will only amplify a non-floxed or non-recombined allele. The black reverse primer sits on the 3′ *LoxP* cassette and the forward in the floxed region so will only amplify floxed animals. Neither primer pair will amplify in cre-recombined animals. (**B**) Breeding scheme. *Vasa-Cre* was used to excise the *Tra2b* PE. Male *Vasa-Cre* transgenic mice were mated with female *Tra2bPE*$^{fl/fl}$ mice to obtain *Tra2bPE*$^{fl/+}$;*Vasa-cre* mice. We then mated male *Tra2bPE* $^{fl/+}$;*Vasa-Cre* mice (the floxed allele will be deleted in mature sperm by the *Vasa-Cre* to generate a deletion allele) with female *Tra2bPE*$^{fl/fl}$ mice to generate four possible genotypes. Actual mouse numbers born of each genotype are shown, along with actual and expected frequencies. (**C**) Example of genotyping result by agarose gel electrophoresis. Lane M marker, lanes 1, 2 presence of the *Vasa-Cre* transgene, lanes 3, 4, 5 different *Tra2b* PE alleles as shown in part (**A**). (**D**) Litter sizes of *Tra2b-cPEko* females and wild type females, after crossing with wild type male mice. Individual litter sizes from wild type female mice are shown as black dots, and the mean as a horizontal line. No litters were obtained from *Tra2b-cPEko* female mice (red dot). 3 breeding cages of each cross were maintained until each of the wild type cages had produced a litter.

© The Author(s)

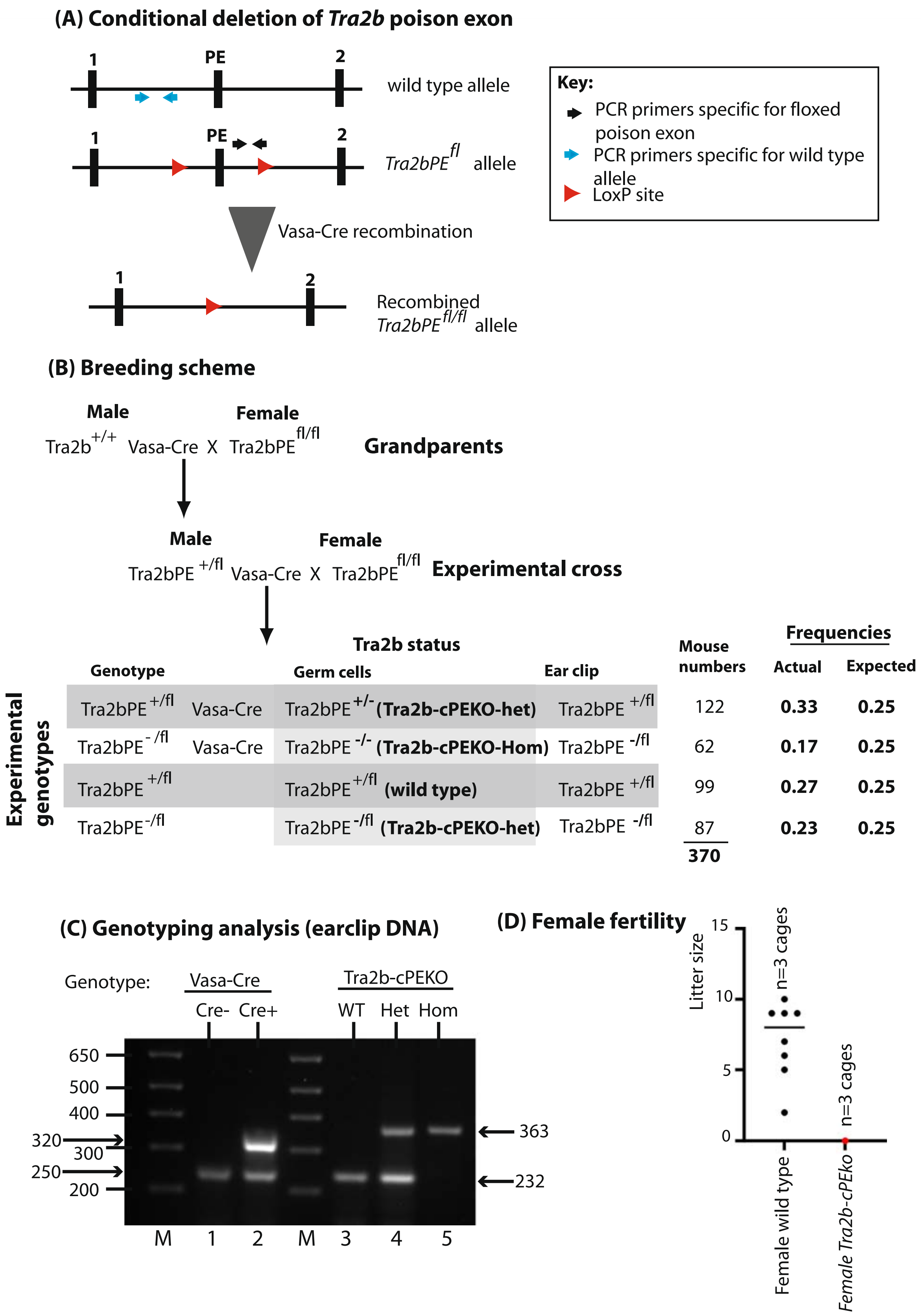
(A) Conditional deletion of Tra2b poison exon
1
PE
2
wild type allele
Tra2bPE fl allele
Vasa-Cre recombination
Recombined Tra2bPE fl/fl allele
Key:
PCR primers specific for floxed poison exon
PCR primers specific for wild type allele
LoxP site
(B) Breeding scheme
Male
Female
Tra2b +/+ Vasa-Cre X Tra2bPE fl/fl
Grandparents
Tra2bPE +/fl Vasa-Cre X Tra2bPE fl/fl
Experimental cross
Experimental genotypes
Genotype
Tra2b status
Germ cells
Ear clip
Mouse numbers
Frequencies
Actual
Expected
Tra2bPE +/fl Vasa-Cre | Tra2bPE +/- (Tra2b-cPEKO-het) | Tra2bPE +/fl | 122 | 0.33 | 0.25
Tra2bPE -/fl Vasa-Cre | Tra2bPE -/- (Tra2b-cPEKO-Hom) | Tra2bPE -/fl | 62 | 0.17 | 0.25
Tra2bPE +/fl | Tra2bPE +/fl (wild type) | Tra2bPE +/fl | 99 | 0.27 | 0.25
Tra2bPE -/fl | Tra2bPE -/fl (Tra2b-cPEKO-het) | Tra2bPE -/fl | 87 | 0.23 | 0.25
370
(C) Genotyping analysis (earclip DNA)
Genotype:
Vasa-Cre
Tra2b-cPEKO
Cre-
Cre+
WT
Het
Hom
650
500
400
320
300
250
200
363
232
M
1
2
M
3
4
5
(D) Female fertility
Litter size
15
10
5
0
n=3 cages
n=3 cages
Female wild type
Female Tra2b-cPEko

 © The Author(s)

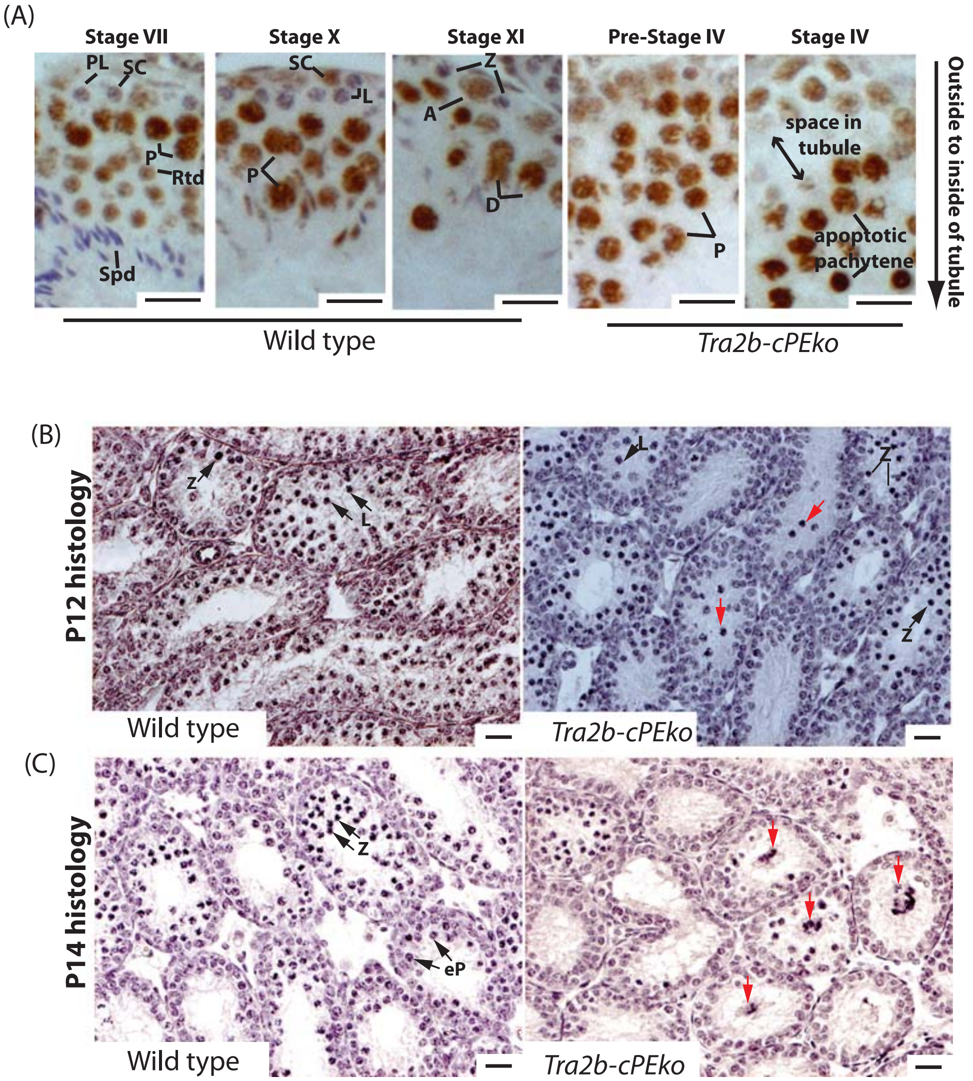

Figure EV2. Analysis of the effect of *Tra2b* PE deletion at days P12 and P14 of mouse testis development.

(**A**) Micrographs showing immunohistochemical detection of Tra2β protein in sections made from wild type and *Tra2b-cPEko* adult mouse testes ($n = 2$). Segments of seminiferous tubules at different stages are shown to enable all the major stages of postnatal mouse spermatogenesis to be visualised. Abbreviations for cell types shown in Fig. 1B, with the addition of SC (Sertoli Cell). Scale bar = 20 μm. (**B**, **C**) Micrographs of Haematoxylin-stained histological sections of (**B**) P12 mouse testes and (**C**) P14 mouse testes of different genotypes. Scale bar = 20 μM. Red arrows indicate sloughing germ cells. Scale bars = 20 μm. Sample numbers for each time point, $n = 4$ wild type and $n = 4$ *Tra2b-cPEko*.

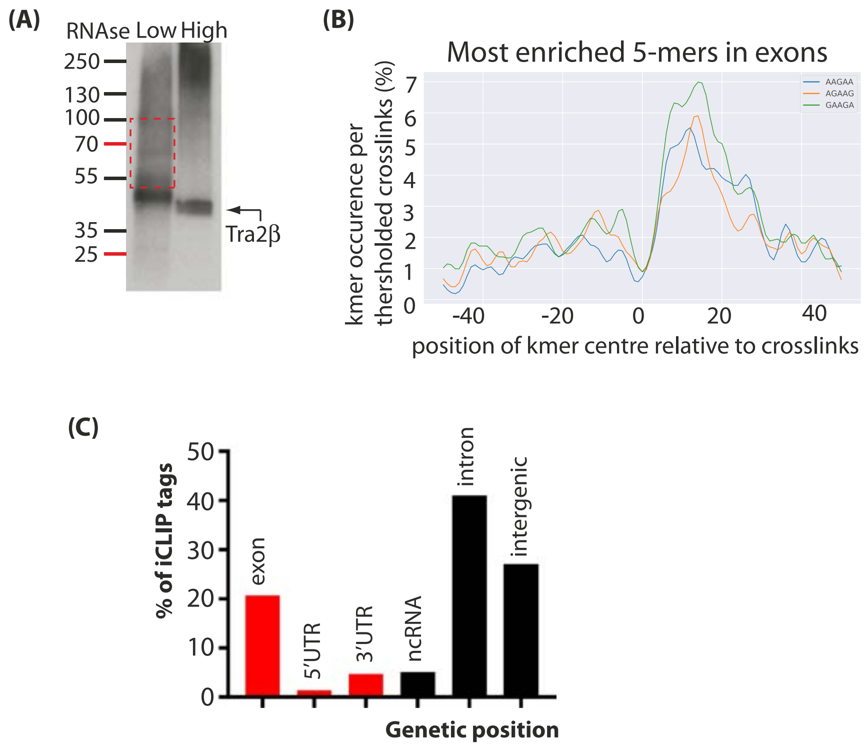

Figure EV3. iCLIP Identification of endogenous binding sites for Tra2β in the adult mouse testis.

(**A**) After cross-linking, endogenous Tra2β protein was immunoprecipitated from wild type mouse testis. Autoradiograph shows 32P-labelled RNA cross-linked to endogenous Tra2β from adult mouse testis during one of three replicate iCLIP experiments. At high RNase concentrations a single radiolabelled RNA-protein adduct of ~40 kDa was detected (arrowed), corresponding to the approximate molecular weight of uncross-linked endogenous Tra2β protein (37 kDa). Tra2β iCLIP tags were recovered at lower RNase concentrations (region used highlighted in red box) in biological triplicate, and these tags were used to map endogenous Tra2β binding sites across the mouse testis transcriptome. (**B**) Analysis of most enriched 5-mers detected close to cross-linking sites within exons (data from one iCLIP replicate shown, similar data were obtained in each of three independent iCLIPs using biological replicate testes). The most frequently occurring pentamers within the iCLIP tags were highly enriched in AGAA nucleotide sequences, exactly corresponding to the known Tra2β binding site (Cléry et al, 2011; Tsuda et al, 2011). (**C**) Genomic distribution of Tra2β binding sites. Average percentage of cross-links from triplicate iCLIP experiments within each genomic region are shown.

 © The Author(s)

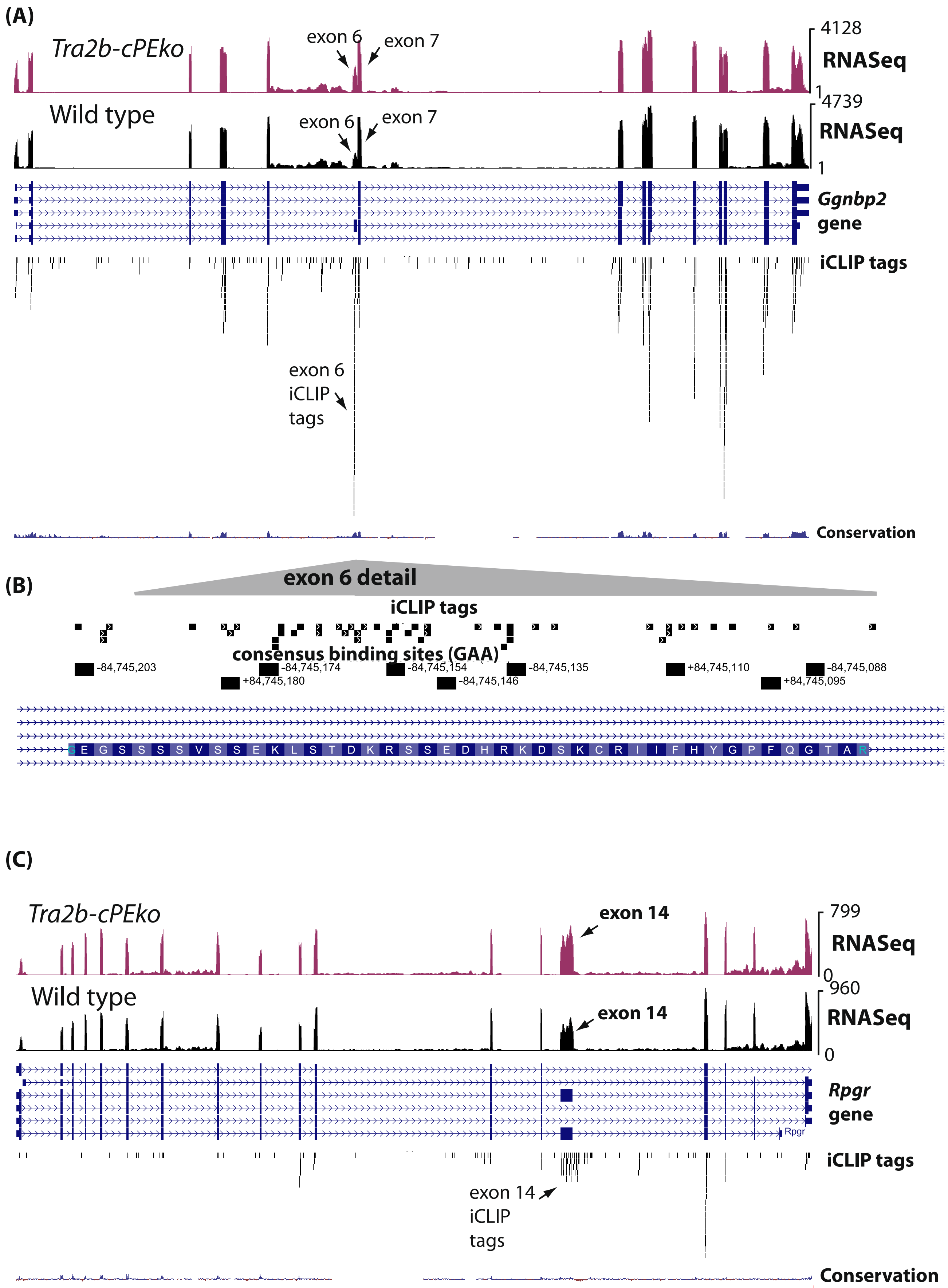
(A)
Tra2b-cPEko
exon 6
exon 7
4128
RNASeq
1
Wild type
exon 6
exon 7
4739
RNASeq
1
Ggnbp2
gene
iCLIP tags
exon 6
iCLIP
tags
Conservation
(B)
exon 6 detail
iCLIP tags
consensus binding sites (GAA)
-84,745,203
+84,745,180
-84,745,174
-84,745,154
-84,745,146
-84,745,135
+84,745,110
+84,745,095
-84,745,088
E G S S S S V S S E K L S T D K R S S E D H R K D S K C R I I F H Y G P F Q G T A
(C)
Tra2b-cPEko
exon 14
799
RNASeq
0
Wild type
exon 14
960
RNASeq
0
Rpgr
gene
Rpgr
iCLIP tags
exon 14
iCLIP
tags
Conservation

Figure EV4. Increased inclusion of Tra2β target exons in the *Tra2bcPEko* mouse testis.

RNAseq reads are merged tracks from testes of 4 P12 mice of different genotypes. Tra2β iCLIP tags are pooled from 3 biological replicate iCLIP experiments using adult wild type mice. (**A**) UCSC mouse genome (mm39) browser screenshot of the *Ggnbp2* gene locus. This screenshot contains the fully expanded iCLIP track, showing an accumulation of iCLIP tags mapping to exon 6. Increased inclusion of *Ggnbp2* exon 6 is detected in *Tra2b-cPEko* mouse testes compared to wild type. (**B**) Detail of exon 6, indicating the positions of experimentally mapped iCLIP tags, and consensus Tra2β protein-RNA binding sites (GAA-containing) sequences. (**C**) UCSC genome browser screenshot showing RNAseq and iCLIP reads aligned to the mouse genome (mm39) at the *Rpgr* locus. Increased inclusion of *Rpgr* exon 14 was detected within the *Tra2b-cPEko* testes compared to wild type.

 © The Author(s)

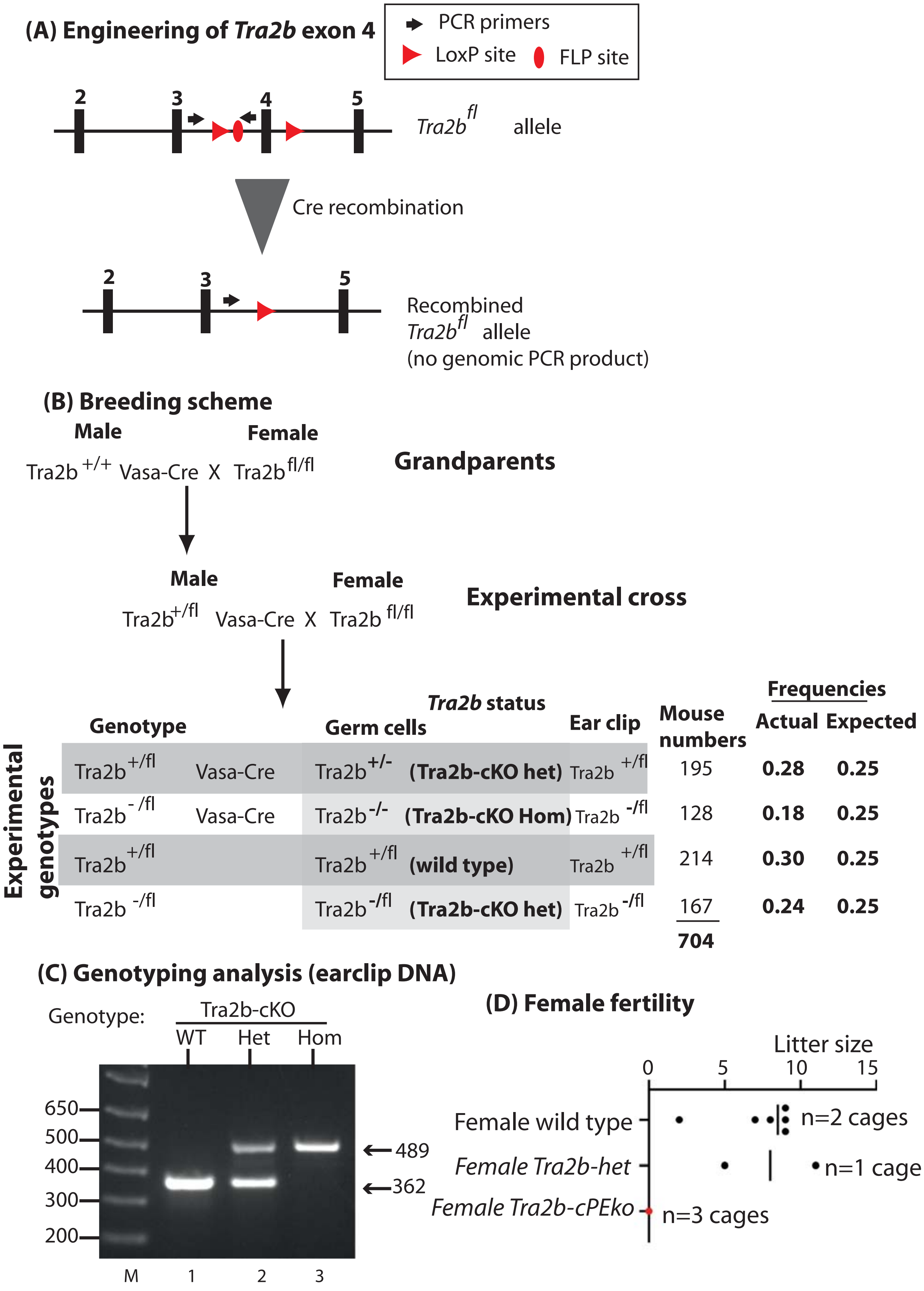
(A) Engineering of Tra2b exon 4
PCR primers
LoxP site
FLP site
2
3
4
5
Tra2b fl allele
Cre recombination
2
3
5
Recombined Tra2b fl allele (no genomic PCR product)
(B) Breeding scheme
Male
Female
Tra2b +/+ Vasa-Cre X Tra2b fl/fl
Grandparents
Male
Female
Tra2b +/fl Vasa-Cre X Tra2b fl/fl
Experimental cross
Experimental genotypes
Genotype
Tra2b status
Germ cells
Ear clip
Mouse numbers
Frequencies
Actual
Expected
Tra2b +/fl Vasa-Cre
Tra2b +/- (Tra2b-cKO het)
Tra2b +/fl
195
0.28
0.25
Tra2b -/fl Vasa-Cre
Tra2b -/- (Tra2b-cKO Hom)
Tra2b -/fl
128
0.18
0.25
Tra2b +/fl
Tra2b +/fl (wild type)
Tra2b +/fl
214
0.30
0.25
Tra2b -/fl
Tra2b -/fl (Tra2b-cKO het)
Tra2b -/fl
167
0.24
0.25
704
(C) Genotyping analysis (earclip DNA)
Genotype:
Tra2b-cKO
WT
Het
Hom
650
500
400
300
200
489
362
M
1
2
3
(D) Female fertility
Litter size
0
5
10
15
Female wild type
n=2 cages
Female Tra2b-het
n=1 cage
Female Tra2b-cPEko
n=3 cages

Figure EV5. Generation of mice with germ cell-specific deletion of *Tra2b* exon 4.

(**A**) An existing conditional *Tra2b*^{fl} allele in which *Tra2b* exon 4 was flanked with LoxP sites was used to inactivate *Tra2b* gene function. (**B**) Experimental crosses. Male *Vasa-Cre* transgenic mice were mated with female *Tra2b*$^{fl/fl}$ mice to obtain *Tra2b*$^{fl/+}$;*Vasa-cre* mice. We then mated male *Tra2b*$^{fl/+}$;*Vasa-Cre* mice (the floxed allele will be deleted in mature sperm by the *Vasa-Cre* to generate a *Tra2b* knockout allele) with female *Tra2b*$^{fl/fl}$ mice to generate four possible genotypes. Actual mouse numbers born of each genotype are shown, along with actual and expected frequencies. (**C**) Example of agarose gel electrophoresis genotyping result. Lane M marker, lanes 1, 2, 3 distinguish the different *Tra2b* alleles shown in part (**A**). (**D**) Litter sizes of *Tra2b-cko* females, *Tra2b-het* and wild type females, after crossing with wild type male mice. Individual litter sizes from wild type female mice are shown as black dots, and the mean as a horizontal line. No litters were obtained from *Tra2b-cko* female mice (red dot). Breeding cages of each cross were maintained until each of the wild type cages had produced a litter.

 © The Author(s)
